# Complex circular subsidence structures in tephra deposited on large blocks of ice: Varða tuff cone, Öræfajökull, Iceland

**DOI:** 10.1007/s00445-016-1048-x

**Published:** 2016-08-01

**Authors:** J. L. Smellie, A. J. Walker, D. W. McGarvie, R. Burgess

**Affiliations:** 1 Department of Geology, University of Leicester, Leicester, LE1 7RH UK; 2 School of Earth, Atmospheric and Environmental Sciences, University of Manchester, Manchester, M13 9PL UK; 3 Department of Earth Sciences, The Open University, Milton Keynes, MK7 6AA UK

**Keywords:** Ice-melt subsidence, Lapilli tuff, Tuff cone, Hofsfjall, Subglacial, Jökulhlaup, Kettle hole, Eemian, Holocene

## Abstract

Several broadly circular structures up to 16 m in diameter, into which higher strata have sagged and locally collapsed, are present in a tephra outcrop on southwest Öræfajökull, southern Iceland. The tephra was sourced in a nearby basaltic tuff cone at Varða. The structures have not previously been described in tuff cones, and they probably formed by the melting out of large buried blocks of ice emplaced during a preceding jökulhlaup that may have been triggered by a subglacial eruption within the Öræfajökull ice cap. They are named ice-melt subsidence structures, and they are analogous to kettle holes that are commonly found in proglacial sandurs and some lahars sourced in ice-clad volcanoes. The internal structure is better exposed in the Varða examples because of an absence of fluvial infilling and reworking, and erosion of the outcrop to reveal the deeper geometry. The ice-melt subsidence structures at Varða are a proxy for buried ice. They are the only known evidence for a subglacial eruption and associated jökulhlaup that created the ice blocks. The recognition of such structures elsewhere will be useful in reconstructing more complete regional volcanic histories as well as for identifying ice-proximal settings during palaeoenvironmental investigations.

**Electronic supplementary material:**

The online version of this article (doi:10.1007/s00445-016-1048-x) contains supplementary material, which is available to authorized users.

## Introduction

**Fig. 1:**
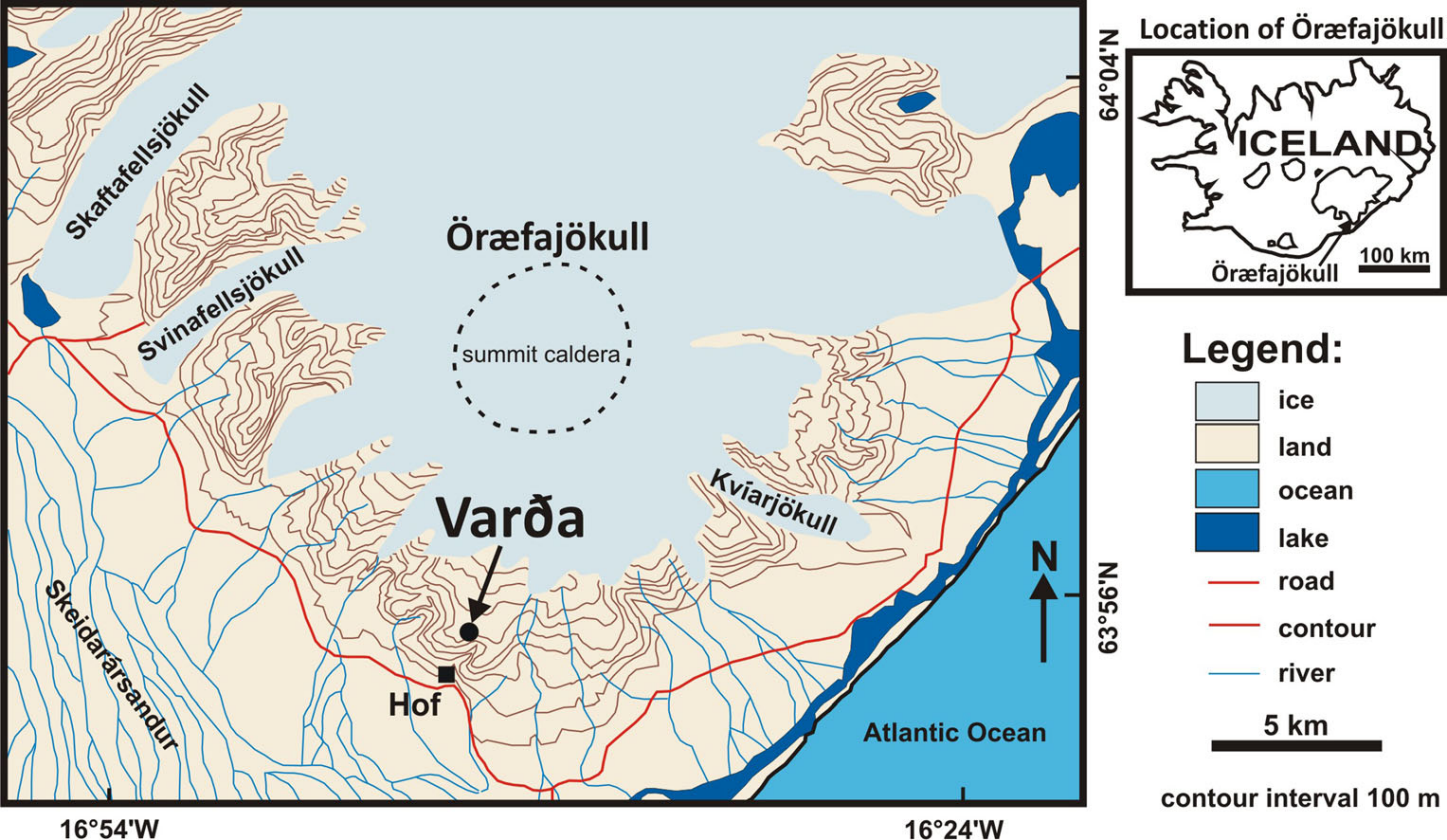
Sketch map of Öræfajökull with the location of Varða indicated. The inset shows the location of Öræfajökull in SE Iceland. Modified after Stevenson et al. (2006)

**Fig. 2:**
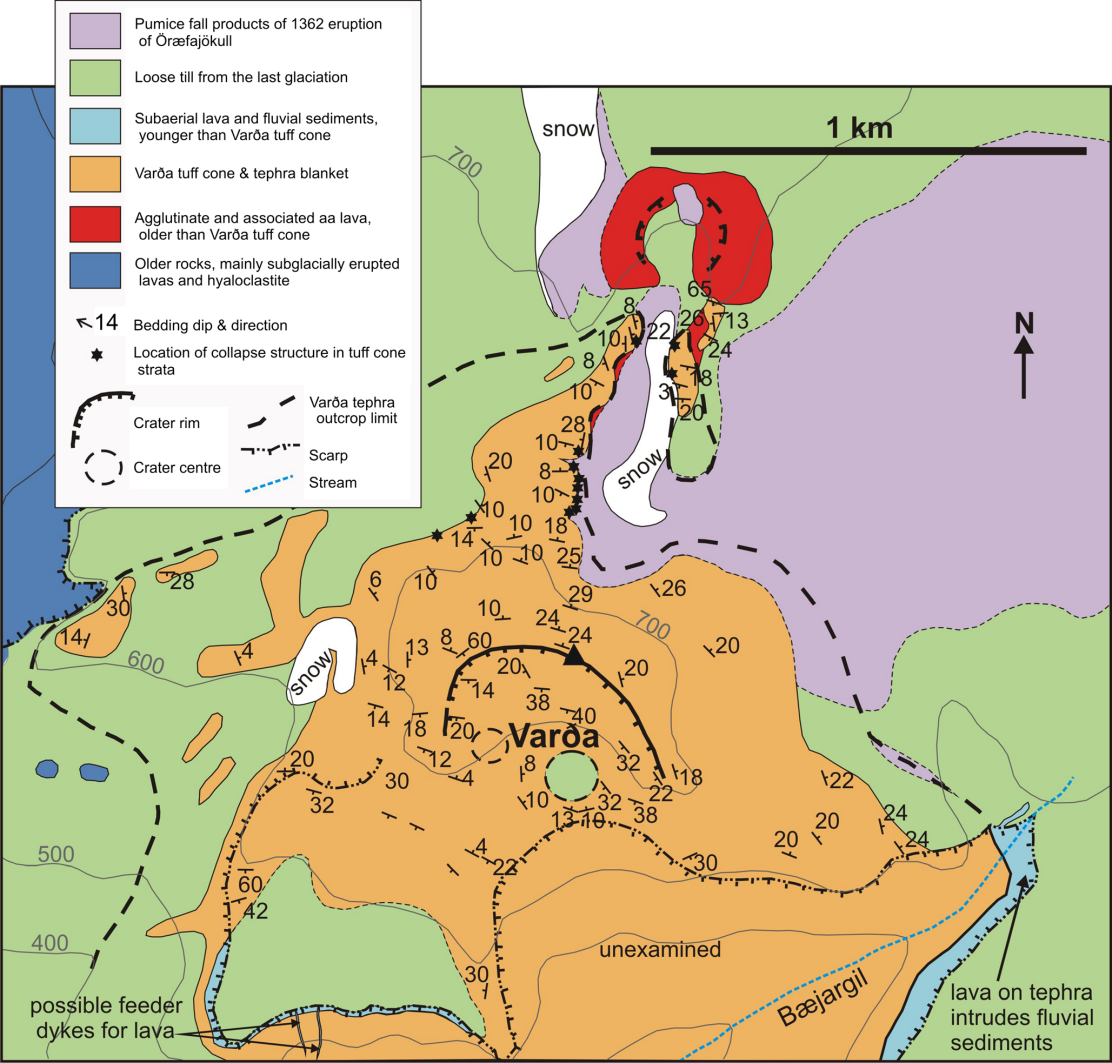
Geological map of Varða. The lava depicted on the southwest flank of Varða appears to be dyke-fed and may be associated with a subsidiary vent of the Varða outcrop

**Fig. 3:**
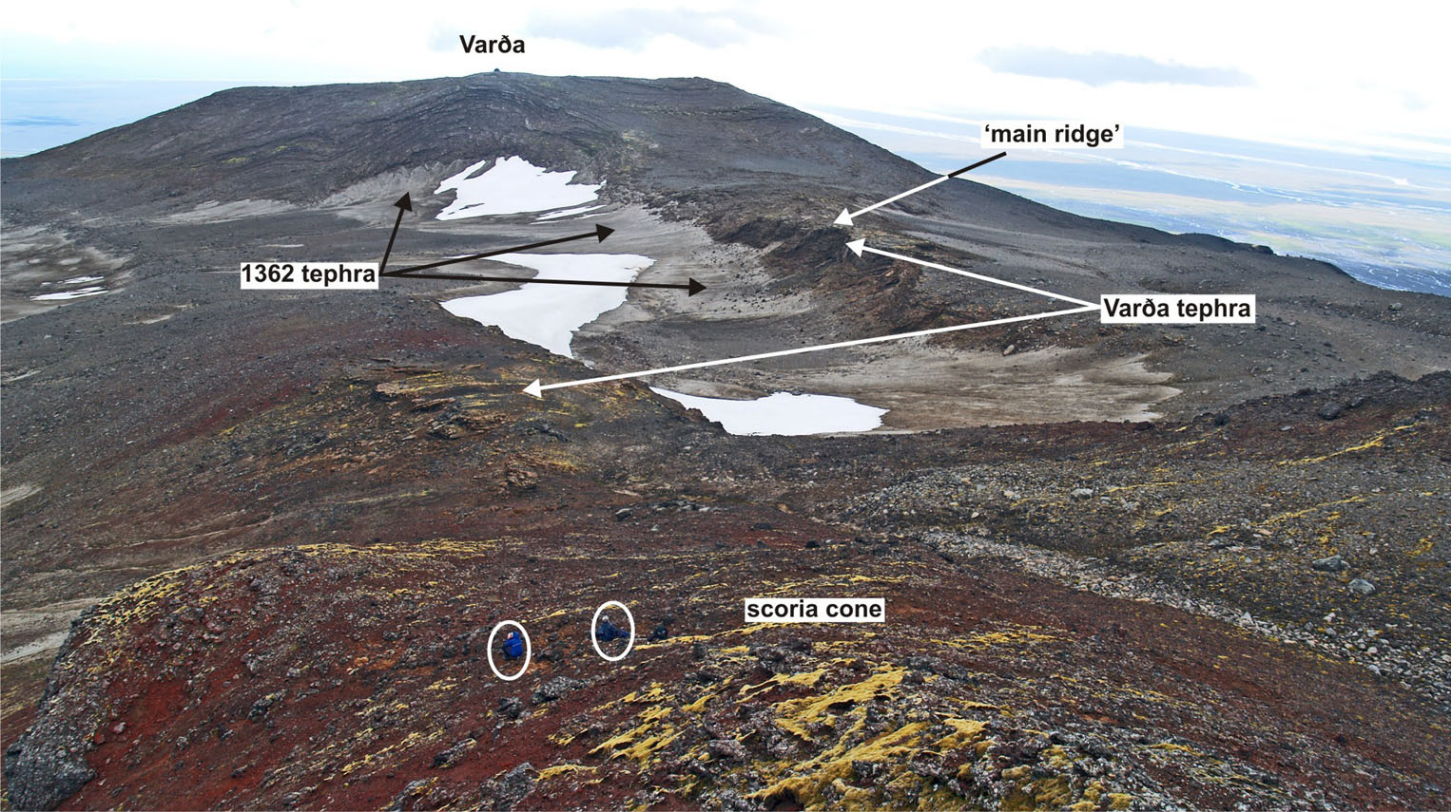
View of the Varða tuff cone looking south from the summit of the small agglutinate cone c. 1 km north of Varða. The shallow valley in the middle ground is covered in pumice tephra from the 1362 eruption of Öræfajökull. Note the topography of the middle ground overlain by Varða tephra. It forms a broad watershed sloping down to right and left. The location of the ‘main ridge’ described in the text is also indicated. Two seated persons in the foreground (*white rings*) are present for scale

Öræfajökull is the largest active stratovolcano in Iceland. It is situated on the southern margin of Vatnajökull and has a basal diameter of c. 23 km, rising to 2110 m a.s.l. at Hvannadalshnúkur a rhyolite lava dome, which is the highest peak in Iceland (Fig. 1). The volcano sustains a prominent summit ice cap that feeds several glaciers on its west, south and east flanks, and it includes a caldera 4–5 km in diameter containing ice up to 550 m thick (Magnússon et al. 2012; Roberts and Gudmundsson 2015). Öræfajökull has erupted large quantities of tholeiitic basaltic rocks together with rhyolites; rocks with intermediate compositions are much less common (Prestvik 1979, 1985). Many of the volcanic products were erupted subglacially (Prestvik 1979; Stevenson et al. 2006; Walker 2011; Forbes et al. 2014). Öræfajökull is known to have erupted twice in historical times: in 1362 and 1727–1728. The eruption in 1362 was the most notable, with ejection of large volumes of rhyolite pumiceous tephra (Thorarinson 1958; Sharma et al. 2008). It devastated a large settled farmed region that was then deserted for more than 40 years and which became known as Öræfi, or wasteland. Both historical eruptions were associated with significant glacier outburst floods, or jökulhlaups (Thorarinson 1958; Roberts and Gudmundsson 2015). Varða (called Hofsfjall in previous publications) is a small hill situated above 600 m a.s.l. on the south flank of Öræfajökull. It is a basaltic tuff cone with a basal diameter of c. 1 km that rises about 70 m above the surrounding landscape. The tuff cone has an associated tephra apron with an outcrop that extends to the north (Figs. 2 and 3). Although the Varða tuff cone has been known for some time (Thorarinson 1958; Wadge et al. 1969; Prestvik 1979), no detailed description has been published. From its dissection and field relationships, it was suggested that the Varða tuff cone was ‘many thousands of years old’ and erupted before the last glaciation (Thorarinson 1958; Wadge et al. 1969).

This paper describes distinctive features in the tephra apron, comprising broadly circular structures up to 16 m in diameter in which higher strata have sagged and locally collapsed. The structures appear to have formed as a result of collapsing into voids. They show evidence for the soft-state deformation of tephras. Soft-state deformation features are commonly observed in pyroclastic deposits of tuff cones, tuff rings and tephra aprons (e.g. Russell and Brisbin 1990; Branney and Kokelaar 1994; Sohn and Park 2005; Zanon et al. 2009; Sohn et al. 2012; Okubo 2014; Vitale and Isaia 2014). When interpreted, they are regarded as instabilities formed due to settling, slumping and subsidence in the edifice or associated with caldera-related volcano-tectonism. However, the structures at Varða are found hundreds of metres away from the coeval edifice, and there is no possible association with calderas. Structures comparable in size and geometry have not previously been observed in tuff cone deposits. Our study suggests that they formed due to the melting out of large buried masses, probably composed of ice. They are thus proxies for a glacial or periglacial setting. Because many volcanoes across the world currently have some ice cover, or had some once, the structures described in this paper are another useful reference tool for inferring former glacial and glacial–proximal environments in volcanic settings.

## Composition, field relationships and deposits of the Varða tuff cone

There are no published analyses of the Varða tephra or its constituents, but the abundance of pale brown sideromelane with narrow palagonite-altered rims and phenocrysts of labradorite–andesine and olivine indicate that it is basaltic. The tephra apron associated with the tuff cone extends c. 1 km to the north where the topography rises slowly to merge with the present-day ice cap on Öræfajökull. Despite significant post-eruption erosion, the conical topographical shape of the tuff cone is still preserved, and the upper slopes, crater and crater rim are recognisable. It is well bedded. The outward-dipping stratification wraps around the two southeast-pointing topographically lower crater-rim ridges suggesting that the cone had an original horseshoe shape ‘open’ to the south, and a significantly lower southern rim when it was active, consistent with a predominantly southerly wind direction during eruption. The rim to rim width of the crater is about 500 m, and it is elongated slightly in a WNW–SSE direction. Changes in the attitude of inward-dipping bedding on the west side possibly define a smaller shallower subsidiary crater now largely back-filled with tephra. Together with the slight northwest–southeast asymmetry of the cone edifice, at least two coeval active craters may have been active. Other active vents are possible and may explain the presence of an enigmatic outcrop of subaerial lava occupying the lower ground on the south west flank of Varða, linked to one or more feeder dykes exposed in the crags overlooking Bæjargil (Fig. 2, and Supplementary Material Fig. 1).

Thick deposits of the tuff cone are well exposed on the steep to subvertical sides of the Bæjargil gorge, and they also drape the steep face of a marine cliff on its south west side (Wadge et al. 1969; Supplementary Material Fig. 1). The deposits are overlain on the south east side of Bæjargil by a younger lava and fluvial sediments; the lava has irregularly intruded and baked the latter. Both the lava and sediments are overlain by boulder till from the last glaciation and they are extensively eroded (Thorarinson 1958; Wadge et al. 1969). The gorge of Bæjargil itself appears to be a postglacial feature that attests to significant fluvial erosion of Varða tephra. The Varða tuff cone is also draped patchily by boulder till almost to its summit and till boulders have accumulated preferentially in the crater bottom (Fig. 2).

The Varða tephras are lapilli tuffs (classification of White and Houghton 2006). The abundant sideromelane ash grains and lapilli are generally ≤5 mm in diameter, pale brown with mainly blocky angular shapes (less often cuspate) and variably vesicular (mainly incipient to moderate, rarely high; sensu Houghton and Wilson 1989), with relatively few small phenocrysts. The proportion of ash is high (typically c. 60 %). Tachylite is also common but less so than the sideromelane. It contains the same phenocryst phases as the sideromelane and is thus petrologically similar and also juvenile. Accessory (lithic) clasts are ubiquitous and comprise angular, non-vesicular petrologically variable lavas typically 1–4 cm in diameter (up to 70 cm) that vary in their grain size (aphanitic to fine grained), colour (shades of grey; rarely red) and phenocryst types and abundance; they are much less common than sideromelane.

**Fig. 4:**
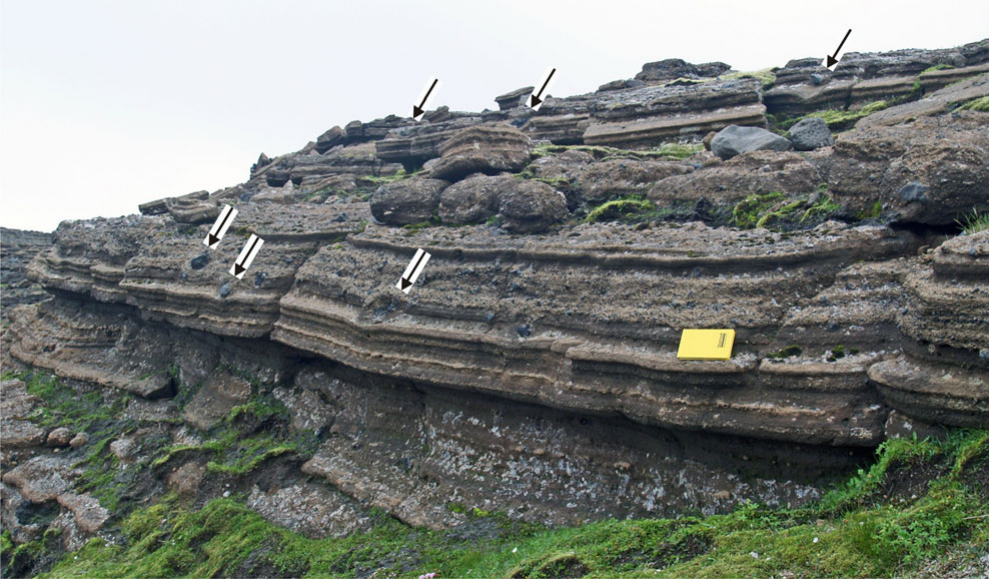
Typical exposure of lapilli tuffs in the Varða tuff cone, showing prominent planar diffuse stratification. *Arrows* mark the positions of outsize clasts with impact structures; ballistic travel from upper right to lower left. The field notebook is 17 cm long

**Fig. 5:**
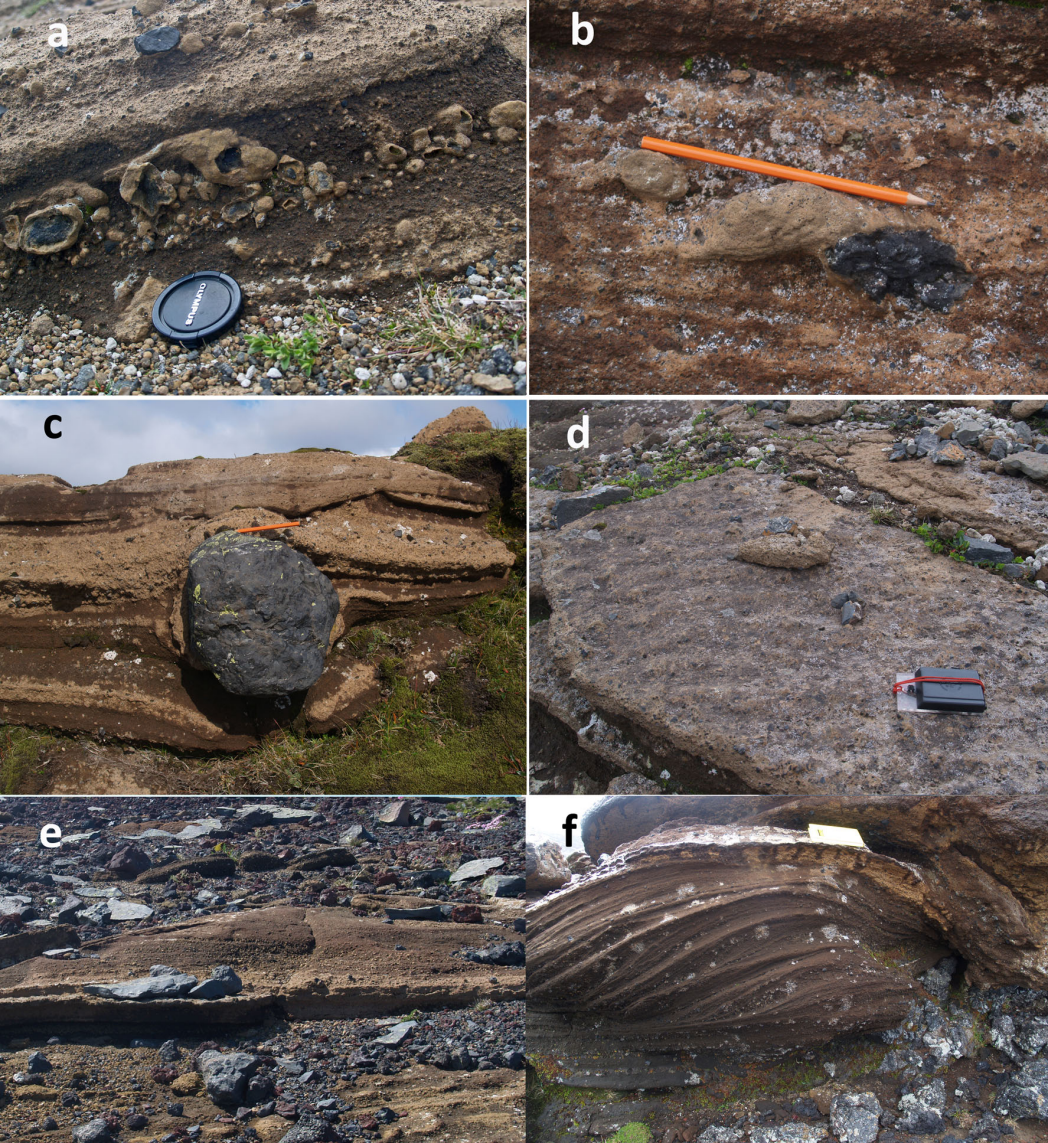
Selected views of features of lapilli tuffs in the Varða tuff cone (**a**–**d**) and off-cone outcrops to the north (**e**–**f**). **a** Abundant armoured (*ash*-*coated*) lapilli; **b** fine tuff (*below pencil*) banked against bomb lapillus (*dark grey*) by a pyroclastic density current travelling left to right; **c** block with impact structure; **d** adhesion ripples; **e** dune bedform with low-angle foresets (transport left to right); and **f** stacked antidunes (transport left to right)

The deposits are diffusely stratified and less commonly thinly bedded (terminology after Branney and Kokelaar 2002; Fig. 4). Thicker (up to 0.5 m) massive beds are more rarely present, some showing coarse-fraction normal grading of lithic clasts. Beds are laterally discontinuous and seldom extend laterally more than a few tens of metres down-dip (typically <30 m); the thinner stratification usually extends just a few metres before wedging out. Armoured (ash-coated) lapilli (sensu Ryane et al. 2011) up to 4 cm in diameter (usually 0.5–1 cm; Fig. 5) are common and conspicuous throughout. Black, highly vesicular glassy juvenile bombs are uncommon and most outsize clasts are lithic blocks. Impact structures are abundant (Fig. 4) and outsize clasts with ramps of fine tuff on the vent-side, adhesion ripples and vesiculation in the fine ash matrix were also observed (Fig. 5). Impact structures are rarely filled preferentially by coarser lapilli. A few dune and antidune bedforms are present c. 800–900 m north–northeast of the crater (Fig. 5); they were not observed in the main tuff cone. Evidence for edifice instability is relatively common particularly on and close to the crater rim. It comprises normal and reverse faults of uncertain but probably small displacement present both within the crater and on the outer slopes, mainly close to the crater rim or cutting obliquely across it. Some are overlain by younger beds. Steeply dipping slipped beds are present within the crater, deformed into small open folds with a wavelength of c. 0.5 m.

**Fig. 6:**
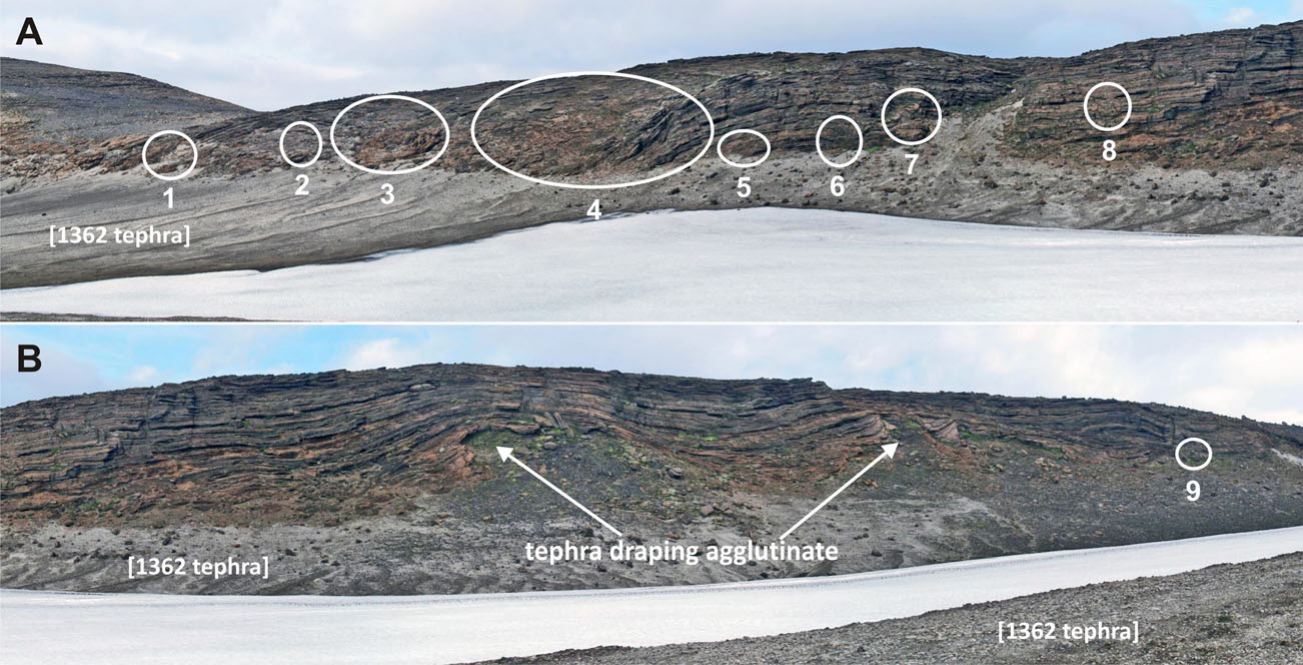
View looking west at the east side of the main ridge extending north from Varða. The locations of ice-melt subsidence structures are indicated and they are numbered to link with descriptions in the text. Note also the two antiforms created by tephra draping older agglutinate mounds (representing a bisected crater rim). The pale scree is composed of 1362 tephra. The cliff face is up to 16 m high

The lapilli tuffs are banked up steeply (65°) at the base of the southern flank of a small pyroclastic cone 900 m to the north (Fig. 2) that is constructed of oxidised agglutinate and they conformably overlie a thin ‘a‘ā lava that flowed south for a short distance out of the agglutinate cone. The agglutinate cone shows poor glacial striations locally. Pristine clinkers derived from the ‘a‘ā lava are dispersed within the basal beds of lapilli tuff nearby. The lapilli tuffs also overlie two small mounds of grey agglutinate (probably representing the crater rim of a second small agglutinate cone transected by the cliff) that are poorly exposed at the base of the cliffs half way along the main north ridge (Fig. 6).

## Eruption character and palaeoenvironment of the Varða tuff cone

The discontinuous stratification, abundance of ash (including fine ash), variably vesicular juvenile lapilli with blocky shapes, abundant impact structures as well as clasts lacking impact structures, together with the presence of dune and antidune bedforms and impact structures filled by large lapilli are characteristic of deposits formed during phreatomagmatic eruptions (e.g. Sohn 1996; Cole et al. 2001; Branney and Kokelaar 2002). They were probably deposited mainly from fully dilute pyroclastic density currents but the poorly sorted massive beds may have been deposited from granular fluid-based currents (cf. lithofacies dsLT, dbLT and mLT of Branney and Kokelaar 2002). The presence of numerous impact structures indicates a ballistic origin for many of the outsize blocks but no evidence was observed for any of the beds having an origin solely by tephra fall. However, the abundance of armoured lapilli, thought to form in a predominantly subaerial moist ash-rich eruption column by processes similar to ash aggregates (e.g. Gilbert and Lane 1994; Brown et al. 2012), suggests that a high proportion of the clasts may have been incorporated by falling into the moving density currents (Brown et al. 2010). Their abundance and a lack of evidence for deposition in water (e.g. no ripples, fossils or sharply defined beds; and abundant impact structures at all levels in the cone) also suggest that the eruption was substantially or entirely subaerial. In addition to the armoured lapilli, the presence of abundant well-formed impact structures, bombs with ramps of fine tuff on the vent-side (i.e. up-current), adhesion ripples and vesiculation in the fine ash matrix (Figs. 4 and 5) all point to a wet, sticky, relatively cool eruptive and depositional environment. Beds dipping at 65° (i.e. well beyond the angle of repose for cohesionless granular materials) at the foot of the scoria cone to the north are further evidence for cohesiveness of the deposits. The Varða tuff cone is thus a hydromagmatic centre in which eruptions were very violent (to generate the large volume of ash observed) and involved magma interacting with water.

There is a lack of any feasible topography that might have impounded a pluvial lake at Varða, and the elevation is too high (>600 m a.s.l.) for the area to have been flooded by the sea. The tuff cone may have erupted either in a surface lake caused by melting through an extensive ice cover or else the supply of water was from a free-flowing aquifer (e.g. highly permeable ‘a‘ā lava autobreccias; Sohn 1996); a thick blanketing cover of snow would not, in itself, be capable of supplying a sufficiently sustained supply of meltwater and would soon become exhausted. An absence of any surrounding ice is suggested by the extensive distribution of the Varða tephra outcrop, including on the relatively steep slopes on the west side below the cone (Fig. 2); any tephra deposited on ice would either be advected away by subsequent glacier flow or disrupted in situ and washed away when that ice underwent static mass wasting and sapping (e.g. on a watershed with negligible ice flow; see later). Furthermore, pristine-looking clinkers derived from the surface of the small ‘a‘ā lava sourced in the agglutinate cone c. 1 km north of Varða were detached and incorporated as dispersed clasts locally in the basal deposits of the overlying lapilli tuffs. This signifies that (1) they were not overridden and abraded by ice prior to the deposition of the Varða tephra (whereas the agglutinate cone itself was, probably at the same time as the Varða cone was overridden) and (2) a thick (several metres) overlying snow or ice cover was not present, as it would have protected the ‘a‘ā lava surface from the effects of the rapidly moving pyroclastic density currents. There is also no evidence for deposition of the tephras in water (e.g. ballistic impact structures are present at all levels in the cone). Thus, it is more likely the water was supplied by an aquifer, consistent with the presence of accessory lithic clasts which would have been derived by explosive detonations within the underlying bedrock. The cone is also located on a high seaward-dipping platform above a cliff. The cliff (best seen just to the east of Hof; Wadge et al. 1969) is draped by Varða tephra (Supplementary Material Fig. 1) and was probably formed by a combination of glacial erosion and, particularly, wave action during interglacial periods of raised sea level similar to the formation of cliffs on the seaward side of Eyjafjallajökull and elsewhere in Iceland (Wadge et al. 1969; Loughlin 2002; Thordarson and Hoskuldsson 2002). Therefore, it is suggested that the magma interacted with groundwater derived from, and easily replenished by, seawater that flowed into cracks within an aquifer. A lack of authigenic zeolites in the pore spaces of the lapilli tuffs prevented the composition of the water (fresh or marine) from being determined (cf. Johnson and Smellie 2007).

## Description of collapse structures at Varða

**Fig. 7:**
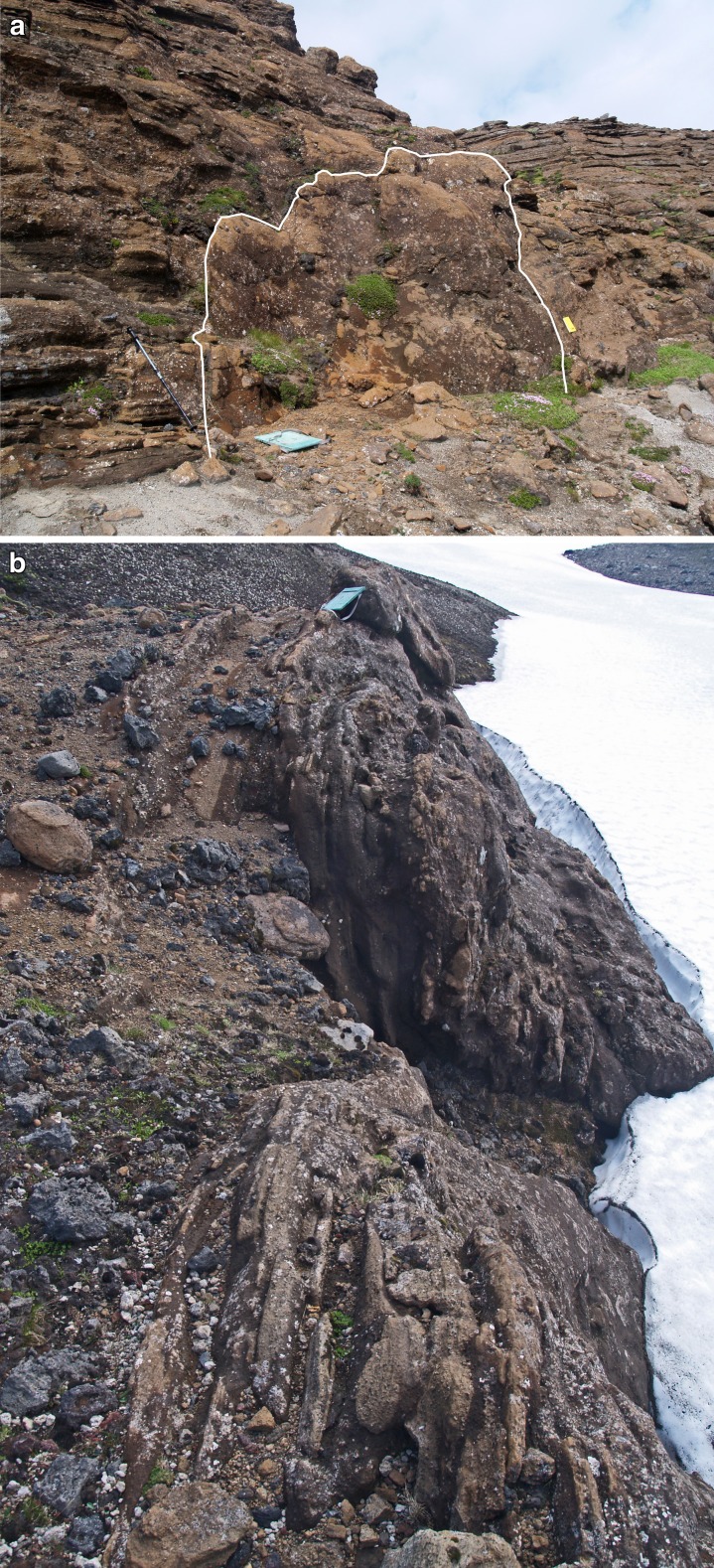
Views of the commonest ice-melt subsidence structures seen at Varða. **a** Steeply-dipping slab of massive lapilli tuff (*white outline*; structure number 6) draped across a subvertical face that cuts across gently dipping stratified lapilli tuff. The notebook is 17 cm in length. **b** Poorly exposed structure on the west flank of the detached Varða tephra outcrop east of the main ridge. The structure comprises a subvertical slab of stratified lapilli tuff a few metres thick. The lapilli tuffs outside (*left*) of the structure dip at 10° and are cut across by the structure (not seen in the photograph). The *green* mapping case is c. 30 cm wide

The low flat-topped ridge extending c. 1 km north of Varða (herein referred to as the main ridge) contains most of the unusual features that are the focus of this study. The tephra forming the ridge was deposited on a pre-existing broad topographical divide, or watershed, as exposed today (Fig. 3). The ridge has an east-facing cliff up to 16 m high in which nine collapse structures are exposed (Fig. 6). Similar but less well-exposed structures are present on its more subdued west flank and also on the west side of the small subsidiary outcrop to the east of the main ridge (Figs. 2 and 3). Of the structures observed, most appear simply to be parts of the margins of structures that have otherwise been completely eroded. They include slab-like sections of stratified to massive fine lapilli tuff up to a few metres thick that dip at c. 80° or greater and truncate bedding in the subjacent lapilli tuffs (Fig. 7). In some cases, the contact with the adjacent lapilli tuffs is slightly overhanging and dips away from the structure itself. The massive lapilli tuff is inhomogeneous, comprising poorly defined domains rich in armoured lapilli or tuff matrix, respectively, together with ill-defined relicts of impersistent stratification in a variety of attitudes. Bedding adjacent to (outside of) the steep slabs is typically sharply truncated but the beds locally dip down within a few decimetres of the contact.

**Fig. 8:**
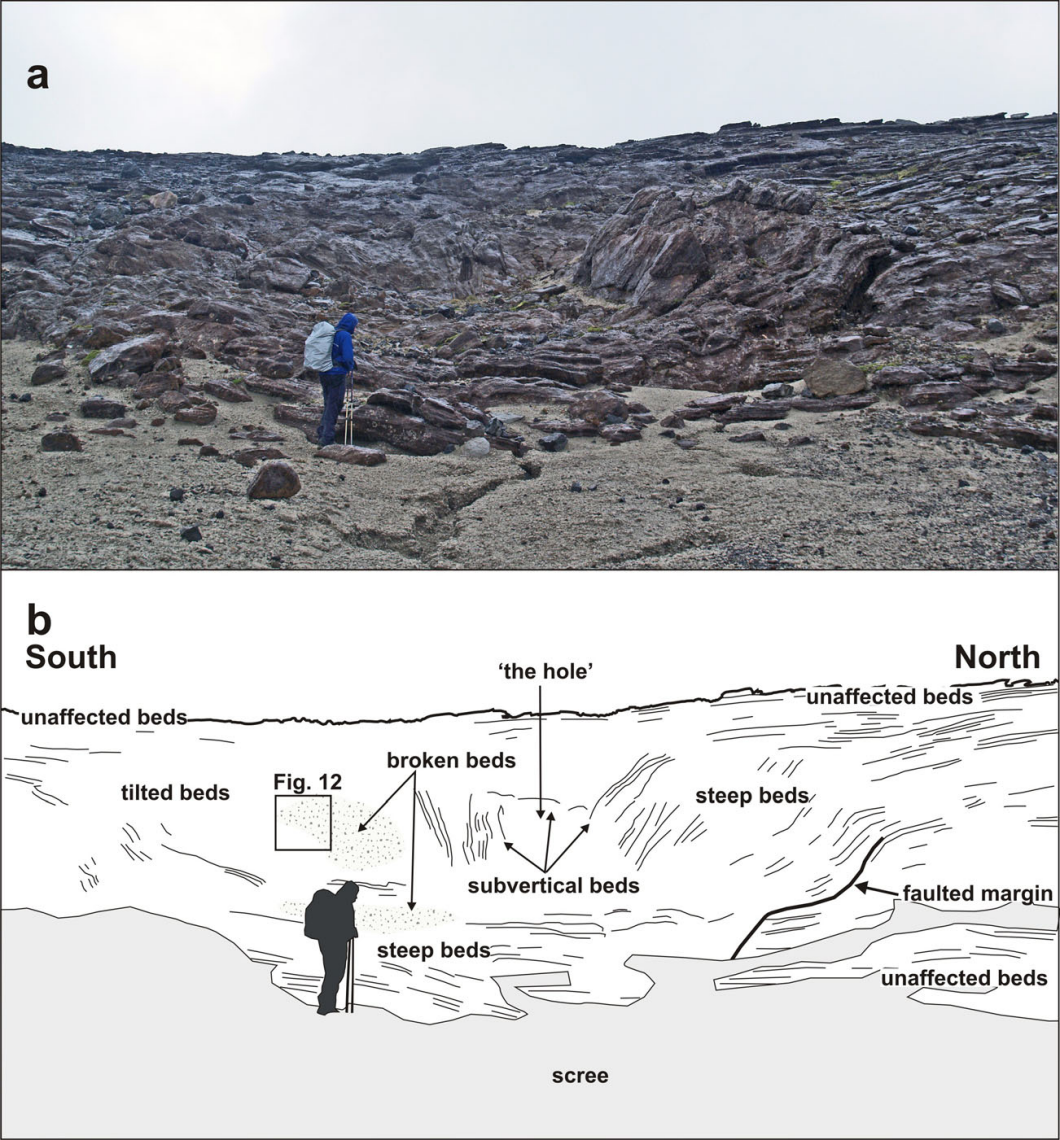
Photograph and annotated view of structure 3, showing steep inward-dipping bedding, which varies to vertical and even slightly overturned, suggesting that a surface pit may have formed as a result of the deformation. The northern margin is faulted. See also Fig. 12

**Fig. 9:**
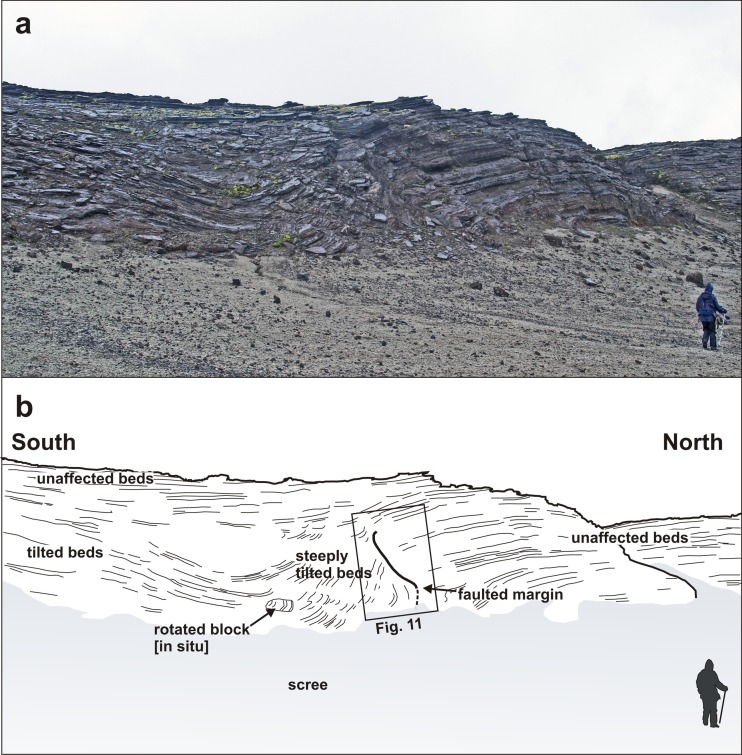
Photograph and annotated view of structure 4. The downsagged strata form an asymmetrical depression, with shallower bedding dips on the south side and much steeper dips on the north side, where there is a prominent faulted margin (shown in Fig. 10)

**Fig. 10:**
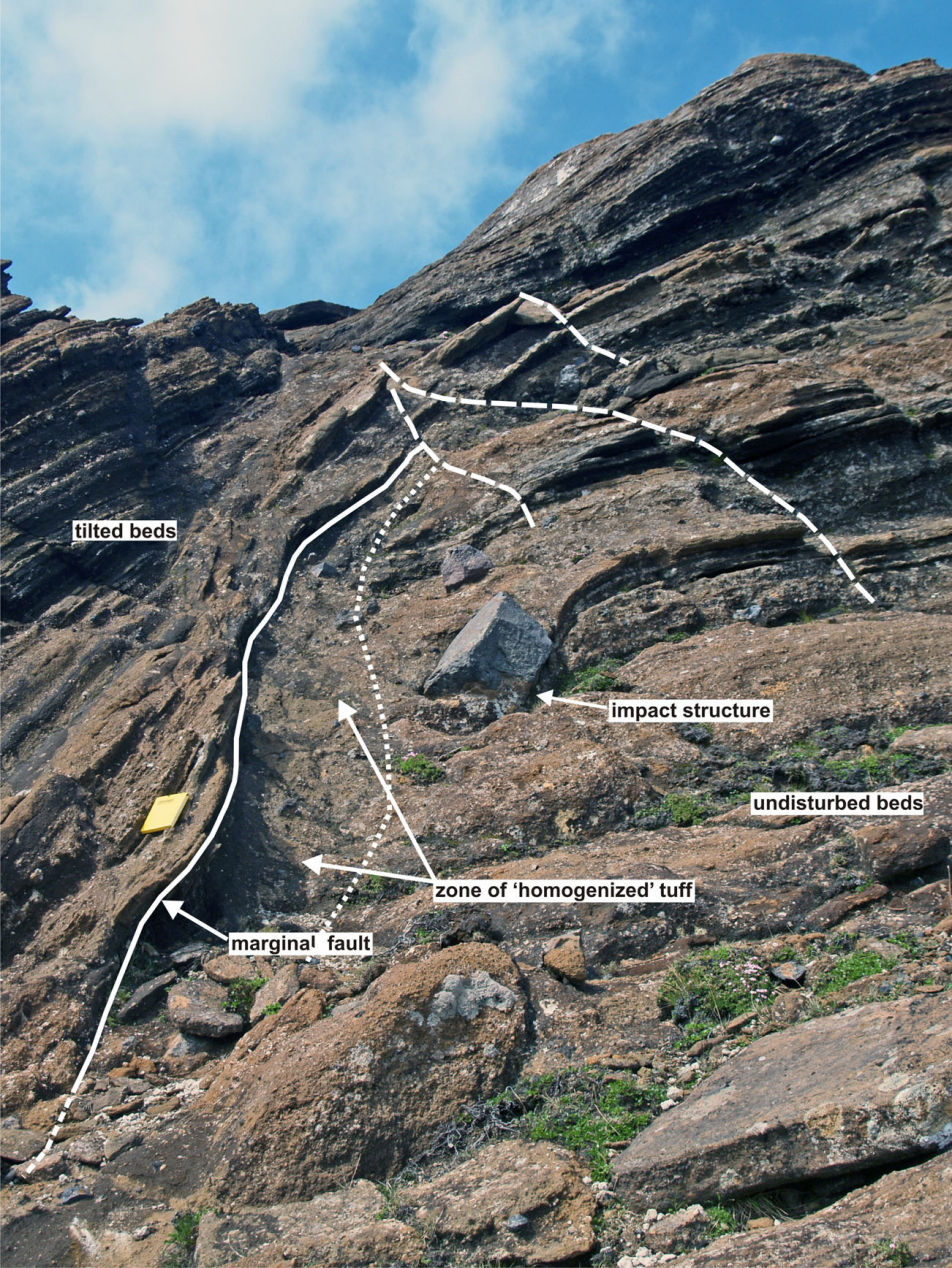
Annotated view of the northern margin of structure 4. The margin is a fault that cuts across lapilli tuff beds that are slightly downbent close to the contact. The relationships suggest that a large block of stratified lapilli tuffs has collapsed along a normal fault and is not exposed at this level whilst the fault is draped by downsagged higher beds. The three normal cross-faults shown (*dashed white lines*) may be coeval with the subsidence structure, and possibly formed due to tension at the axis of flexure for the downsagged beds. The notebook is 17 cm in length

**Fig. 11:**
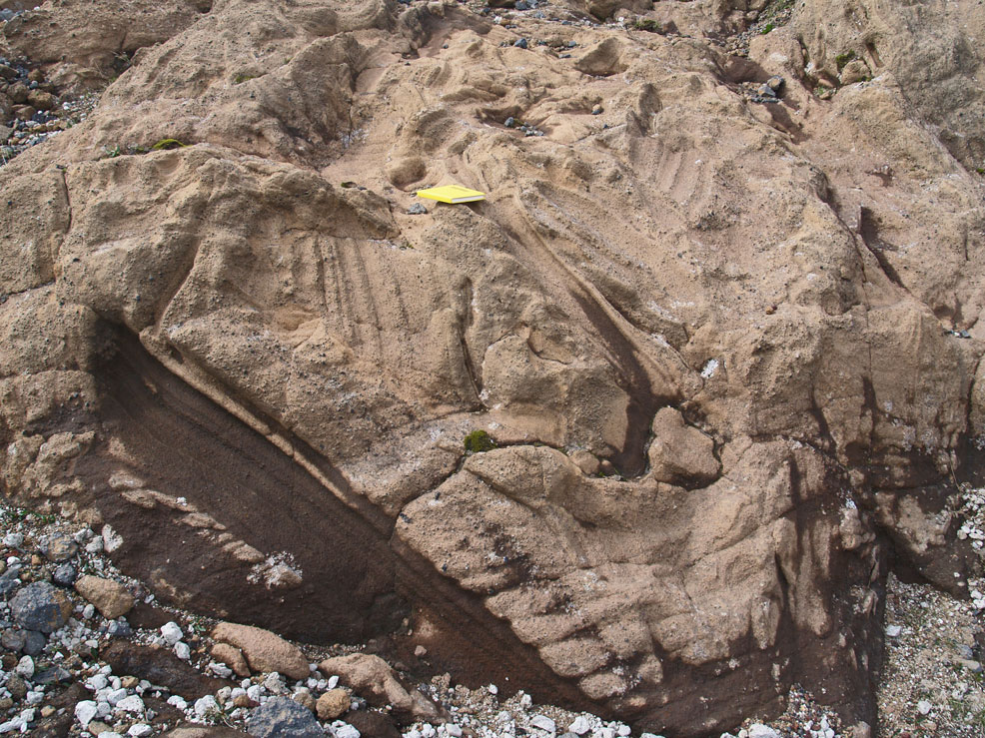
Blocks of stratified lapilli tuff within structure 3. Note the ill-defined margins of the blocks and lack of open pore spaces, which suggests that the blocks were relatively weakly lithified when they were juxtaposed and partially disaggregated during formation of the breccia. The notebook is 17 cm in length

**Fig. 12:**
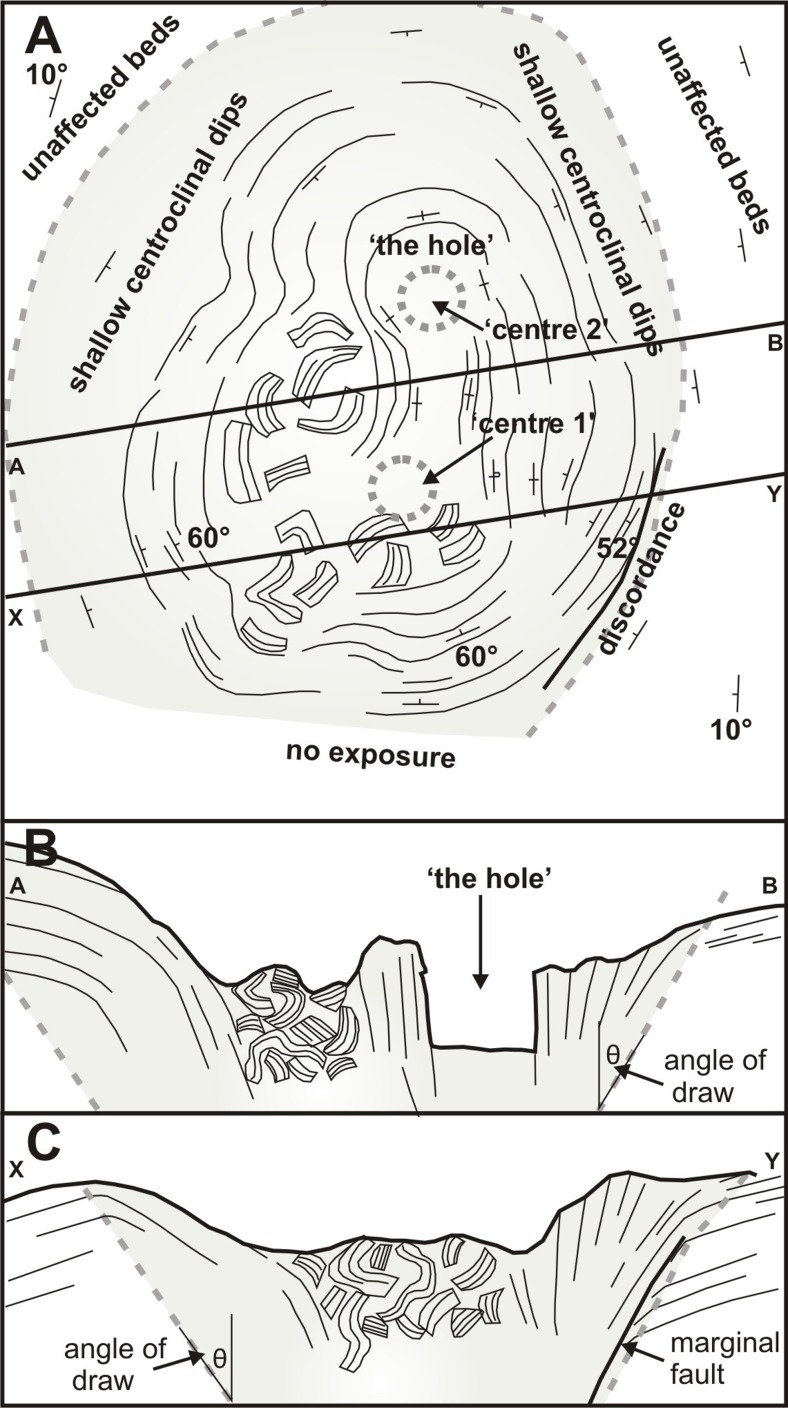
Field sketches of structure number 3. **a** Plan view; **b**, **c** Cross sections along lines shown in **a**. Angle of draw (θ) is the angle at which the subsidence spreads out; it defines the limit for subsidence effects (Whittaker and Reddish 1989; Ren and Li 2008). In **a**, ‘centre 1’ and ‘centre 2’ refer to the locations of two possible buried ice blocks whose mutual melting caused the subsidence structure to have an elongated, ‘figure of eight’ configuration (see text for details). Note the presence of breccia sandwiched between tilted beds in **b**, suggesting that subsidence was probably sequential (piecemeal)

Two of the structures (3 and 4 in Fig. 6) are more completely preserved than the others (Figs. 8 and 9). The structures are of similar size, broadly circular and measuring c. 13–15 m N–S and c. 12–16 m E–W but the east flanks are truncated by erosion and they may be elliptical overall. Both are exhumed to c. 8 m in depth but their bases are unexposed. The upper beds within each structure appear to have sagged down by c. 3 m or so. The structures are asymmetrical internally, with inward-dipping downsagged strata on all sides but one side (northern in both cases) that includes a fault-like discordance (Figs. 8, 9 and 10). In structure 3, strata rapidly increase their centroclinal dip to 60° as they enter the structure, becoming vertical and locally even slightly overturned further within the structure interior. In addition to downsagged strata, the southern half of structure 3 includes polygonal blocks of stratified lapilli tuff juxtaposed at all angles (Fig. 11). It also contains an approximately circular present-day pit c. 3 m in diameter (called ‘the hole’ in Fig. 8) surrounded on three sides by vertical beds (Figs. 8 and 12); the fourth (eastern) side is eroded. Structure 4 is dominated by more gently downsagged strata with dips mainly increasing only to c. 20° but dipping more steeply inward at the discordant northerly margin similar to structure 3 (Figs. 9 and 10). Its surface therefore resembles a shallow asymmetrical depression rather than a steep-sided pit. Beds outside both structures dip at 8–10° to the south, reflecting the regional gradient of the pre-eruption landscape, which rises to the north (Fig. 2). The rare appearance in structure 4 of a displaced metre-long block of strata with steep-dipping bedding suggests that some beds have also been fragmented, similar to structure 3 but less well developed (i.e. fewer blocks) at the level of exposure seen in structure 4. A thin (30–40 cm wide) irregular zone of massive lapilli tuff is developed locally along the steeply inclined contact on the northern margin of structure 4 (Fig. 10). The sharp contact is cross-cutting and fault-like lower down where it truncates beds. Small normal and reverse faults with centimetre- to decimetre-scale displacements are common in the overlying downsagged beds but without consistent orientations. Faults are uncommonly present in the beds outside all of these structures, but they are not obviously related geometrically to the concentric structures. Folded and displaced bedding is also prominent enclosed in massive lapilli tuff in the smaller structure 7 (c. 3 m in diameter).

## Discussion

### Origin of the Varða collapse structures

Important features of the structures include (1) faulted steep-dipping (vertical to slightly overturned) margins that sharply crosscut external lapilli tuff strata; (2) downbending of some beds directly adjacent to the faulted margins; (3) local presence of a marginal zone of massive lapilli tuff with a semi-homogenised appearance, within which are indistinct blocks of stratified beds; (4) the higher beds simply bend down into the structure (rather than becoming thicker, e.g. infilling a depression), defining symmetrical and asymmetrical concentric sag-like geometries; and (5) some structures contain breccia composed of stratified blocks with bedding in various dips. A lack of burial by younger tephra suggests that the structures formed after the eruption had ceased. Moreover, distinctive sedimentary structures would have formed in pyroclastic density current deposits passing over surface hollows, with variations in bed thickness and grain size.

The surfaces between adjacent blocks in the breccias are ill-defined (Fig. 11) and the presence of narrow zones of homogenised lapilli tuff at the margins of several structures (Figs. 7a and 10), which contain poorly seen stratified blocks preserved in a massive matrix, suggest that the lapilli tuff was still weakly lithified when it fragmented to form breccias and underwent soft-state granular fragmentation when stressed. The absence of voids between clasts probably indicates infilling by grains generated by disaggregation, and further reduction in void space may have been caused by compression and ductile folding of some clasts during compaction. The downward transfer of material, by tumbling, granular flow and viscous creep, is called suffusion. It is well known in sinkholes and dolines, where it is usually caused by rainwater gradually washing unconsolidated material into cavities (Waltham et al. 2005). Some of the faulted margins are slightly overhanging, suggesting that the adjacent ash-rich beds outside of the structures were cohesive enough to preserve a vertical face. The overlying beds were also cohesive but reacted plastically, locally sagging down into a void to rest against steep underlying marginal fault surfaces (Figs. 10 and 12c). In some cases, beds may have become detached and slipped down as slabs, causing the marginal shear effects observed (bending and bed homogenization). Detachment may have been progressive and sequential rather than a single-stage process (as was also postulated for the kettle holes in Mt. Hudson lahars; Branney and Gilbert 1995). This is suggested by the observation that coarse breccias formed of stratified blocks are occasionally sandwiched between tilted beds, implying a sequence of tilting, brecciation and further tilting (Fig. 12b). Slabs of tephra, on a range of scales from metres to decimetres in thickness, may have sagged down, with some breaking off and sliding intact whilst others were fragmented. The polygonal slabs of lapilli tuff are thus interpreted as have accumulated by dropping into a void, as the structure worked its way up to the surface (cf. Whittaker and Reddish 1989).

Bed strength would have been greatly weakened by any coeval fractures caused by settling in the tephra pile as it accumulated, and they are perhaps represented by the intersecting tensile fractures, some with decimetre displacements, observed in the higher downsagged tephra beds. Collapsing beds that remained intact as stratified slabs may have been more cohesive or been deformed at lower strain rates, perhaps related to more gradual mass withdrawal due to slower melting or the local geometry of the melting ice. The ubiquitous evidence for sagging suggests that the overall circular structural features are collapse structures. Analogue models suggest that collapse rates during subsidence vary temporally and spatially, and are associated with incremental accelerations (e.g. Poppe et al. 2015). Compared with the gentle dips elsewhere in the surrounding outcrop (Fig. 2), the collapse structures at Varða are highly localised and suggest that large buried masses were removed in situ from beneath the tephra pile at several locations. There is no preserved material to show what the buried masses consisted of as the bases of the Varða structures are unexposed.

### Nature of the buried material

Options for the buried material that melted below the Varða tephra and caused its localised collapse include (1) a thick snow cover, (2) snow drifts (sastrugi), (3) dirt cones, (4) deep bedrock depressions filled by snow and (5) ice blocks. These are examined further below.

How tephra subsides when it covers a thick layer of snow or firn is hard to predict. Tephra fall deposits with grain sizes comparable to those at Varða are completely cold on deposition (e.g. Thomas and Sparks 1992) and any underlying snow will be effectively protected by a tephra layer >50 cm thick (Manville et al. 2000; Brock et al. 2007). Under such a thick layer, melting would be very slow. For example, snow >0.5 m thick is still preserved under tephra from the 1875 eruption of Askja (Carey et al. 2010, Fig. 3b, and personal observation of M Branney). By contrast, deposits of pyroclastic density currents, such as characterise the Varða outcrops, can have significant temperatures (up to c. 400 °C; Sulpizio et al. 1998; Zanella et al. 1998), which might enhance the melting of any underlying snow or ice. In some cases, phreatic explosion pits can be created in pyroclastic density current deposits, as occurred following the 1980 Mt. St Helens eruption (pits 5–100 m wide and 1–20 m deep; Rowley et al. 1981). However, the Varða structures lack evidence for blast excavation and associated ejecta deposits and an explosive origin is highly unlikely. This suggests that the Varða deposits were much cooler on deposition and unable to flash water to steam. The temperature in these water-rich so-called wet surges was less than 100 °C and any steam present would be condensed to water droplets (Druitt 1996). Temperatures will be lowered further (to ambient) after deposition directly onto snow or ice simply by the energy transferred to melt the snow or ice surface. The thermal conductivity of lapilli ash is so low that further melting will be very slow, although the actual melting rate is unknown. In the Askja example cited, ≥2 m of dilute density current deposits locally overlie as little as 50 cm of tephra fall that rests directly on the coeval snow layer (Carey et al. 2010). The heat from the density current deposits was clearly unable to pass through the fall layer to melt the underlying snow.

The authors are unaware of published studies of the mechanical response of a thick layer of tephra (of any kind) resting on a melting layer of snow but some localised circular collapse structures with steeply tilted beds and pits have developed below the 1875 tephra at Askja (although with smaller diameters than at Varða and forming prominent lines of pits; personal observations of M Branney). However, there is evidence that thick snow (i.e. more than a few metres) was probably absent at Varða when the tephra was deposited (see above). If the melting layer was isotropic (i.e. had a constant thickness and uniform grain size), we speculate that subsidence of an overlying thick tephra layer might be relatively uniform, with pits restricted to anisotropic patches of snow, i.e. with a different crystal structure or hardness. If the snow layer was highly anisotropic (e.g. uneven in thickness, perhaps reworked by wind into hard-packed drifts (sastrugi) prior to tephra deposition), then melting might over time lead to differential subsidence. However, sastrugi are typically ridge-like with sharp, linear, wavy or otherwise sculpted crests and sloping often asymmetrical flanks, and occur in fields of closely spaced landforms. Sastrugi are also generally smaller, seldom more than a few metres high and wide. Whether their melting beneath a thick tephra layer might result in isolated concentric collapse pits with vertical to slightly overhanging margins (as are typical for the Varða structures) is unclear as there are no described examples.

Pits might also form by the melting of the snow cores of buried dirt cones. Dirt cones in volcanic regions like Iceland commonly form by the differential melting of snow beneath a surface ash layer of variable thickness; they are particularly common on glacier ice. Although dirt cones are usually <1–3 m high, exceptionally they can be much larger, up to 85 m (Swithinbank 1950; Krenek 1958; Hauff 1969), thus overlapping in size with the Varða structures. Dirt cones occur in close-spaced clusters or lines and are short lived, normally lasting just a few weeks. Like melting buried sastrugi, there are no published examples of structures resulting from melting of buried dirt cones, and it is unclear whether large steep-sided pits like those at Varða will be created. However, their natural occurrence in close-spaced groups should probably result in moundy terrain rather than the isolated collapse pits seen at Varða. Moreover, there is no evidence that the Varða eruption was glaciovolcanic since ash deposited on glacier ice will be rapidly advected away. Undisturbed tephra (lacking collapse structures) crop out in situ resting on bedrock on the relatively steep western slope below the tuff cone, consistent with an absence of ice around Varða during eruption.

Collapse into deep bedrock depressions, such as a concealed tectonic fissure, lava tubes or tumuli, is also discounted. No such features were observed in the surrounding area (the lavas are ‘a‘ā, not pāhoehoe) and, even if such features are unexposed, there would be lithofacies evidence, such as bed thickening and preferential deposition of coarser clasts. They are thus unlikely to be involved.

The collapse of sediment into voids created by the melting of buried ice is plausible and is our favoured option. Similar surface structures on glacial outwash plains (sandurs) are known as kettle holes. Kettle holes are hollows created in the sedimentary deposits when partially to completely buried blocks of glacier ice melt out (e.g. Maizels 1977). They are common in the proglacial sandur areas of Iceland, and their widespread presence and large sizes are diagnostic of glacier outburst floods, or jökulhlaups (McDonald and Shilts 1975; Maizels 1977, 1992; Fay 2002a, b; Marren 2005; Russell et al. 2006; Roberts and Gudmundsson 2015). As such, they can be used to identify palaeojökulhlaups in the geological record (Marren 2005). The associated ice blocks can be a few tens of metres in diameter and height (e.g. Burke et al. 2010), and the larger ones are usually not completely buried (Supplementary Material Fig. 2). Kettle holes become filled mainly by stratified fluvial sands and gravels (Olszewski and Weckwerth 1999). The sedimentary layering in the infill is centroclinal and it may be folded, faulted or partly fragmented. Vertical contacts between the infill and exterior sediments are also rarely present. These features are distinct from the Varða structures, which exhibit only deformation rather than sedimentation into a pit. The internal features of kettles caused by subsidence are not commonly described but consist of brittle fractures, which are mainly outward-dipping reverse ring fractures (McDonald and Shilts 1975; Maizels 1977, 1992; Cocksedge 1983; cf. Branney and Gilbert 1995). A key difference is the sequence of events envisaged for kettle holes versus the Varða structures, i.e. ‘conventional’ sandur kettle holes are formed by ice block deposition during a sediment-laden flood, whereas the scenario envisaged for the Varða structures comprises a discrete phase of ice block deposition, followed by tephra deposition, and then ice block melting.

The dimensions of the Varða structures (c. 16 m in width) can be used to estimate the approximate dimensions of the former buried ice blocks, taking into account that the subsided volume will flare upwards at the angle of draw (θ), thus widening the diameter of the structure at the surface (Whittaker and Reddish 1989; Branney 1995). The angle of draw is typically taken as c. 35° but it varies between c. 10° and 50° depending on the rheology (strength) of the subsiding strata (Ren and Li 2008). Weak strata such as unconsolidated sands and clays, which are probably most comparable with ash-rich lapilli tuffs, tend to have higher θ values (c. 35–40°). Using a mean value of 38° yields a possible width of c. 8 m (range c. 7.5–9 m for θ = 35–40°) for an ice block with a horizontal upper surface buried to a depth of c. 5 m; calculated diameters become greater at lower values of θ (i.e. steeper angles of draw) whilst deeper burial will reduce the apparent width. A burial depth of 5 m was used in the calculations because the observed surface sag of 3 m is consistent with a total subsidence depth of 5 m (i.e. after bulking effects are removed; see next section) and the observation that collapses over buried voids propagate vertically upwards before flaring out near the surface at the angle of draw (e.g. Roche et al. 2000; Acocella 2007; Burchardt and Walter 2009; Howard 2010). Thus, at least some of the buried blocks may have measured 8 m wide and 5 m high. An origin by melting of ice blocks emplaced ballistically is unlikely. The collapse structures occur out to about 800 m from the Varða crater, whereas ballistic lithic blocks 500 m from the crater only reach 1.1 m in diameter; ice blocks of equivalent mass at that distance would have been less than 3 m in width (based on differences in density).

There are also considerable similarities with structures known as ice-melt collapse pits found in deposits of lahars sourced in snow- and ice-clad volcanoes and formed by melting of ice blocks (as at Mt. Hudson; Branney and Gilbert 1995; Table 1). Lahar deposits are often relatively mud rich and therefore more cohesive than sandur sands and gravels. However, although the ice-melt collapse pits at Mt. Hudson are of comparable dimensions (up to 15 m across) to those at Varða, they were preserved in plan view only and there was no erosional dissection. We concur with the mode of origin described by Branney and Gilbert (1995) and many of their interpretations are applicable to the structures which we describe here (Table 1). There are some differences, however. The Varða structures lack arcuate peripheral extensional fractures at the surface and steeply outward-dipping subsurface fractures, which implies that there may be rheological or cohesivity differences with the Mt. Hudson lahar deposits. However, the arcuate extensional crevasses surrounding the Mt. Hudson ice-melt collapse pits would probably have been temporary, in that their steep walls would soon have crumbled/disaggregated and infilled, or become blurred by water oozing up through the deposits and making the sediments ‘quick’. A similar explanation might apply to the structures at Varða. For example, the narrow zones of massive ‘homogenised’ lapilli tuff adjacent to the faulted margins of the Varða structures (Fig. 10) might be escape zones caused by upwelling displaced water disrupting, entraining and mixing material from poorly consolidated strata, i.e. they represent ‘quick’ zones created as the surface sagged and meltwater flowed up arcuate marginal faults. However, the common occurrence of beds outside the structure that are deformed (i.e. physically downbent) and the absence of evidence for surface venting (e.g. as ‘sand volcanoes’) strongly suggest that the massive zones are more plausibly related to marginal shearing during downfaulting. The differences between the ice-melt collapse pits described by Branney and Gilbert (1995) and the Varða structures are probably minor and ascribable mainly to the different stage of evolution of the two occurrences (i.e. more mature at Varða) and possibly greater cohesivity of the Varða deposits. Although surface sagging was probably common, if not ubiquitous, in the Varða examples, it is unclear how often a surface pit was formed (it probably occurred in the case of Varða structure 3), and we prefer to call them ice-melt subsidence structures rather than pits or cavities.

**Table 1:** Ice-melt collapse pits and associated features found in lahars following with the 1991 eruption of Mt. Hudson, Chile (after Branney and Gilbert 1995 and Branney 1995)

Feature	Description	Origin	Comments
Host lithology	Poorly sorted, unconsolidated, mud to boulders (mainly silt to sand) deposits with abundant pumice lapilli and lesser scoria	Debris flow and hyperconcentrated flow deposits composed of a mixture of remobilised 1991 eruption-related fall tephra, older fall tephra and ash-rich glaciofluvial and laharic deposits reworked from the valley floor	Generally less cohesive than the Varða deposits, which are lapilli tuffs formed during hydromagmatic eruptions; the Varda lapilli tuffs are ash-rich and formed cohesive (‘sticky’) deposits capable of deforming ductilely soon after deposition
Obstacle marks	Large shallow scour marks and associated ring-shaped, crescentic and ridge-like gravelly sediment deposits; crescents up to 60 m long; lee-side ridges up to 20 m long and up to 50 cm high	Caused by vortices and other hydrodynamic effects of meltwater currents passing around stranded ice blocks (e.g. Fay 2002b)	Not seen (unexposed) at Varða but possibly present at depth if pyroclastic density currents interacted with stranded ice blocks similar to fluvial current interactions
Dirt cones	Isolated conical mounds of debris 1–3 m in diameter composed of pumiceous and lithic debris dispersed across the lahar surface	Ice-rafted debris left behind after stranded ice blocks on the lahar surface melted out, leaving behind their sediment loads	Not seen at Varða
Kettle holes	Large (25–150 m wide) irregularly shaped pits with near-vertical walls containing water and floating rafts of pumice lapilli	Surface collapse caused by melting of buried ice blocks	Other than their water infill, lapilli rafts and larger dimensions, it is unclear how these differ structurally and in their infill from ice-melt collapse pits
Surface pits	Steep-sided circular surface pits, 1–15 m in diameter and up to 2 m deep; c. 4 pits per 1000 m^3^	Surface feature caused by collapse to form a pit during melting of buried ice blocks; called ice-melt collapse pits; analogous to kettle holes	Present at Varða (e.g. structure 3 and possibly also indicated by isolated exposures of vertical to slightly overhanging lapilli tuff ‘slabs’ (e.g. Fig. 7a))
Circular pit with central subsided sediment block	Small pits ≤1.5 m wide; generally symmetrical; mostly ≤3 m of subsidence; larger examples show signs of downsagging and internal fragmentation	Early immature stage of subsidence	Not seen at Varða but possibly present (too poorly exposed)
Outward-dipping ring fractures	Multiple concentric fractures, sometimes a single fracture, dipping at 25–50°; fractures overhang at surface and rapidly collapse	Reverse faults caused by subsidence into a void at depth	Not seen at Varða but probably present at depth (unexposed)
Trapdoor structures with a monoclinal hinge and horseshoe-shaped fractures	Includes most collapse pits >2 m in diameter; rarely smaller pits; often markedly asymmetrical; includes downsagging; surrounded by convex-out arcuate fractures dipping at <50°; extensional; form on the hinge of the ‘trapdoor’ slab of sediment	Immature pits formed by subsidence in more competent sediment favouring fracturing rather than monoclinal flexuring; more isotropic sediments favour development of larger, more symmetrical features as subsidence progresses	Asymmetrical surface depressions present at Varða (e.g. structure 4); rare normal faults caused by tension at the hinges of some downsagged beds (Fig. 10)
Downsagging and encircling circular faults	Saucer-shaped shallow depressions up to 1 m deep with centroclinal dips generally ≤40°; all tilted masses with dips >30° (up to 90°) associated with outward-dipping to vertical ring fractures with no discernible downthrow; up to three concentric ring fractures; moat-like grabens may develop between faults during dilation	Surface dilation of ring fractures caused by peripheral extension during downsagging	Downsagging locally well developed at Varða (structures 3 and 4; Figs. 8 and 9))
Topographical embayments	Scalloped topographical rims formed around the margins of some larger collapse pits, with recessed embayments up to one third of the pit diameter; some associated with subsided polygonal blocks	Gravitational collapse of steep and over-steepened fault surfaces	Not seen at Varða
Polygonal blocks and avalanche debris	Juxtaposed blocks with subvertical, concave and convex sides; avalanche debris is associated with surface fractures having steep or overhanging walls	Form where intersecting sets of arcuate fractures developed during piecemeal collapse after downsagging; pits >2–4 m deep contain tilted marginal blocks that slid and tumbled down, some disaggregating in the process; avalanching occurs during collapse of unstable surface faults evolving to more gravitationally stable configuration; the avalanche debris forms lobes or aprons that obscure the internal details of the pits, which acquire a funnel shape	Present internally at Varða (Figs. 11 and 12)
Funnel-shaped pits	Depressions with centroclinal surfaces formed of disaggregated sediments	Formed by avalanching of disaggregated sediments during collapse of unstable subaerially exposed fault surfaces	Not seen; Varða examples more cohesive than Hudson sediments, hindering en masse disaggregation
Aqueous modification	Incised pit margins and infilling of pits by rainwater	Rim erosion by surface wash; undercutting and degradation of steep margins by waves	Not seen at Varða; local climate very different?
Diameter to depth ratios of subsidence pits	3:1 to 20:1	Depth is a measure of the distance from the rim to the floor observed in subsidence pits	Poorly known for Varða structures; approximately 5:1 for structure 4

### Comparison with other geological collapse structures

The collapse structures at Varða also show a resemblance to simple downsag structures found in calderas, despite the differences in scale (cf. Branney 1995). They form due to subsidence involving inward tilting or rotation of strata with or without accompanying faulting. There are numerous experimental and field-based studies of the formation of calderas and they provide insights into the likely mode of formation of the Varða structures.

The most important factors governing subsidence mechanisms during caldera collapse are the mechanical and geometrical properties of the overburden, in particular its strength or cohesion, and the roof aspect ratio (AR, i.e. ratio of roof thickness to width of the depression; e.g. Whitaker and Reddish 1989; Roche et al. 2000, 2001; Burchardt and Walter 2009; Holohan et al. 2011; Poppe et al. 2015). Sagging is enhanced in low-strength roof-rocks and at low AR; brittle piston-like subsidence along ring faults is favoured by moderate roof-rock strengths and intermediate AR; and stoping by high roof-rock strengths and at high AR (Roche et al. 2000, 2001; Holohan et al. 2011). Near-continuous collapse (tilting) is associated with a gradually depleting subsurface mass, whereas near-instantaneous collapse (fragmentation) occurs associated with a subsurface metastable cavity (e.g. Poppe et al. 2015).

Experimental studies suggest that two types of ring fractures are generated: an inner set of steeply-dipping, outward-inclined reverse ring faults and an outer set of inward-dipping normal faults (e.g. Roche et al. 2000; Acocella et al. 2000, 2007; Walter and Troll 2001; Geyer et al. 2006; Martí et al. 2008; Burchardt and Walter 2009; Supplementary Material Fig. 3). Since the collapses are induced by a loss of support at depth, the ring faults nucleate there and propagate vertically upwards without significant lateral expansion. As they approach the surface, they flare out and initiate surface tilting inward followed by faulting (normal or reversed according to the fault geometry) when the faults reach the surface. Steep (vertical to slightly overturned) strata (cf. Fig. 7) are often associated with the reverse ring faults (e.g. Poppe et al. 2015). Brittle fragmentation of the roof by caving or stoping may occur during the upward migration of sub-surface cavities formed at the apex of intersecting reverse ring faults (Roche et al. 2001; Acocella 2007). Volumetric expansion of the collapsing roof material (known as ‘bulking’) occurs in both near-instantaneous and near-continuous collapse and it is particularly well developed in roof rocks with high cohesion (Whittaker and Reddish 1989; Poppe et al. 2015). It occurs not only during the upward propagation of reverse ring faults but also during the collapse of the roof rock, and it results in a surface depression that is significantly smaller (by an average of c. 39 ± 11 %) than the space vacated by removal of mass at depth. Asymmetry in the surface expression of subsidence is explained in several ways. Low values of AR (<1) cause asymmetrical ring fault development. The associated collapse is also typically asymmetrical, with maximum subsidence on the side of the first reverse fault (Roche et al. 2000). Subsidence above a cavity with a sloping upper surface will also cause asymmetrical downsagging (Whittaker and Reddish 1989), because collapse will initiate preferentially on the side where the roof thickness is greatest (Roche et al. 2000).

**Fig. 13:**
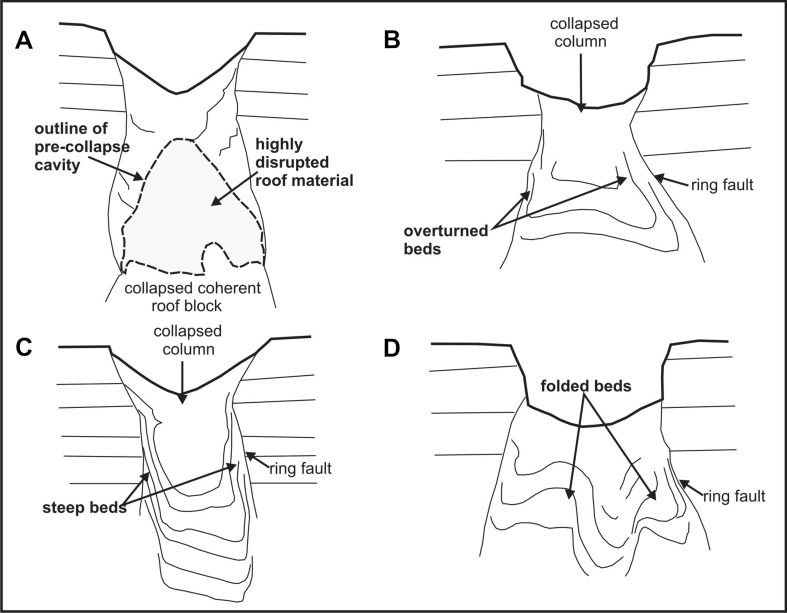
Series of sketches showing internal structures observed in experimental analogues for caldera collapses that are similar to those seen at Varða (based on computed X-ray microtomographic images in Poppe et al. 2015). **a** Breccia development in a former cavity; **b**, **c** steep and slightly overturned beds associated with marginal ring faults; and **d** folded beds within the collapsed mass

Cumulatively, these observations may help to explain some significant features of the structures at Varða. AR values are unknown but, given the measured widths of c. 16 m and a similar maximum thickness estimated from exposures in the main ridge, they are probably close to 1. Some of the structures have asymmetrical surface depressions and normal-faulted margins on one side only (structures 3 and 4; Figs. 8, 9, 10 and 12), implying an AR ≤ 1 or subsidence over a cavity with a sloping upper surface. Moreover, since the greatest void depth will be below the highest point of a buried melting mass, the greatest void space will be created there (assuming a level surface below the ice block) and subsidence will therefore also be greatest, leading to asymmetry in the surface depression. The complicated association of tilted beds and breccia in some structures (cf. Figs. 12b and 13) is consistent with a higher AR or caving and stoping during upward reverse ring fault propagation associated with ephemeral cavity formation and piecemeal subsidence. The observed depths of subsidence in Varða structures 3 and 4, approximately 3 m, are probably minima due to bulking. A ‘full’ subsidence depth of at least 5 m is implied, corresponding empirically to the thickness of the buried ice blocks at those localities. Finally, elongated depressions will form due to structural coalescence caused by the melting of adjacent ice blocks, and this may be an explanation for the ‘figure of eight’ surface morphology of structure number 3 at Varða (cf. ‘nested or dumbbell-shaped pits’ of Branney and Gilbert 1995; Fig. 12).

### Age of the Varða tuff cone and sequence of events

It is noticeable that the ‘a‘ā lava and agglutinate surfaces directly beneath the Varða lapilli tuffs are not glacially eroded but elsewhere they are modified and covered by till. They show no evidence of water or ice interaction and they signify eruption under essentially dry subaerial conditions, i.e. non-glacial and ice free or at least ice- or snow-poor. Moreover, their pristine state suggests that they were erupted not long before the tuff cone (i.e. perhaps by up to a few decades, though probably much less). Although relatively well preserved, apart from the post-glacial carving out of the Bæjargil gorge, the Varða tuff cone is also glacially eroded and it is draped by till from the last glaciation. The agglutinate cone that fed the underlying ‘a‘ā lava also shows signs of overriding ice (local striations). However, ice was not present at the site of either pyroclastic cone during their eruption. An interglacial Eemian age (i.e. c. 132–116 Ka) is thus possible for eruption of both the agglutinate cone and the Varða tuff cone (as suggested by Thorarinson 1958), and the inferred distribution of snow and ice was much reduced compared to that during glacial periods when an ice sheet covered all of Iceland and may have extended out to the shelf edge (Hubbard 2006). However, the presence of ice-melt subsidence structures analogous to fossil kettle holes indicates that ice was present on the landscape not far from Varða. The simplest explanation is that there may have been an ice cap on Öræfajökull, similar to today.

An alternative scenario is for eruptions to have occurred in the Holocene followed by a short-lived advance of the Öræfajökull ice cap to cover and erode the volcanic deposits and leave behind a drape of till. To be viable, the advancing ice must have exceeded 70 m in thickness at Varða (the height of Varða above its surroundings). It could have occurred following the Allerød warm period, when the Icelandic ice sheet expanded greatly at least twice (at c. 10.3 and 9.8 ka BP, e.g. Norðdahl et al. 2008). A Holocene age would be consistent with the moderately well-preserved morphologies of the Varða and agglutinate cones.

The preservation of structures consistent with former buried ice blocks indicates that a jökulhlaup occurred not long prior to the eruption at Varða (e.g. Marren 2005). Varða is situated high up on the flank of Öræfajökull, a large volcano, and Iceland (generally, and including Öræfajökull) is well known for its many volcano-triggered jökulhlaups (e.g. Thorarinson 1958; Roberts and Gudmundsson 2015). It is unknown if the jökulhlaup event that created the ice blocks was related to a subglacial eruption but it is plausible. Other options for catastrophic floods, such as the sudden release of a glacier margin lake or collapse of meltwater impounded by large terminal moraines (e.g. Tweed and Russell 1999), do not fit well with the likely landscape inferred from the present topography. The location of the vent is unknown other than it must have been upslope of Varða and presumably beneath the present Öræfajökull ice cap. The blocks were probably ripped off the ice sheet terminus by the force of the rapidly exiting meltwater, as is commonly observed during modern jökulhlaups, but a supraglacial derivation is also possible and is recorded for historical jökulhlaups sourced on Öræfajökull (Jónsson 1982; Tómasson 1996; Fay 2002a, b; Russell et al. 2006; Roberts and Gudmundsson 2015). Ice blocks transported in jökulhlaups are preferentially deposited on higher ground (Maizels 1992; Fay 2002a; Russell et al. 2006), which may explain their preservation on the shallow watershed now overlain by the tephra outcrop forming the main north ridge; they are absent in the outcrops draping the steeper slopes to the west (Figs. 2 and 3). Ice blocks can survive on the surface without melting typically for only for a few years, although kettle holes (lacking ice) may survive for decades (McDonald and Shilts 1975; Roberts and Gudmundsson 2015). The duration of unmelted buried ice is unknown but it will be enhanced by the insulation caused by the thick layer of Varða tephra, and the local elevation (c. 600 m) and consequent cool ambient temperatures of the site (cf. snow preserved below tephra from Askja erupted in 1875; Carey et al. 2010, Fig. 3b). If the ice blocks postulated by our study were sourced in a subglacial eruption, as seems likely, the ice-melt subsidence structures preserved in the Varða tephra are the only known evidence for that eruption. The glaciovolcanic centre and its products may exist but are covered by the Öræfajökull ice cap.

**Fig. 14:**
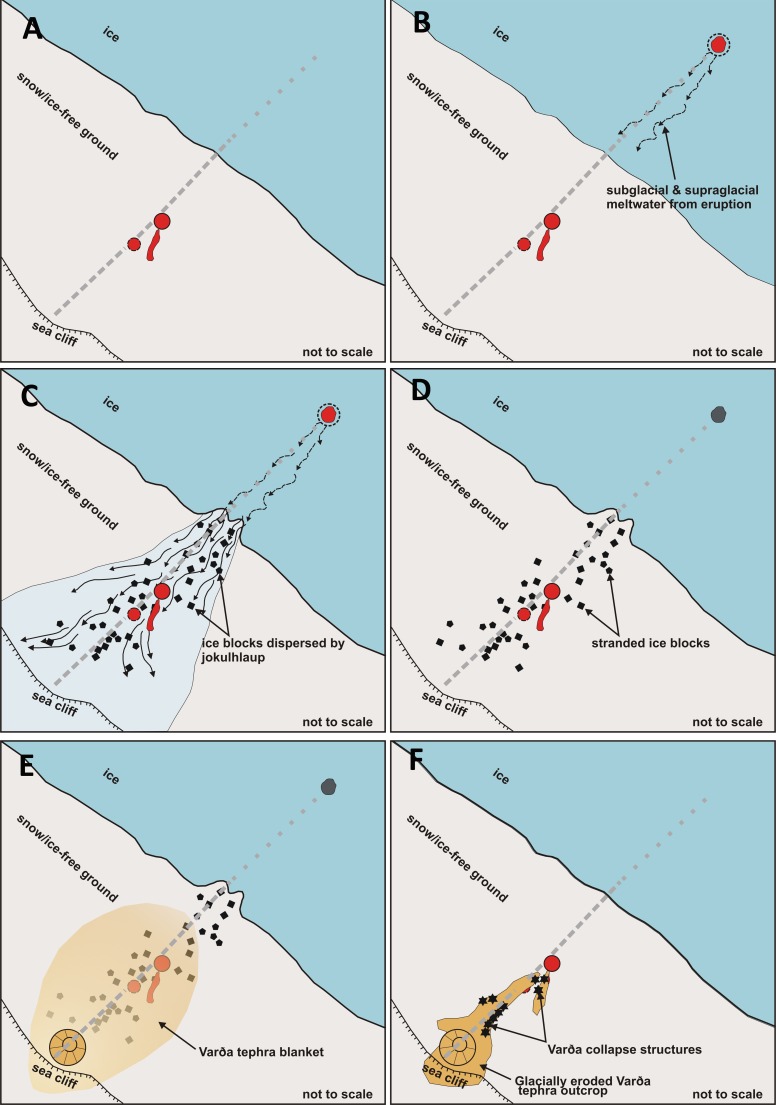
Schematic depiction of the sequence of events envisaged leading to the formation of the Varða ice-melt subsidence structures. The *dashed grey line* in each figure indicates the approximate axis of the broad watershed; it may also correspond to a volcanic fissure, now unexposed and inferred only by the positions of the eruptive centres. The sequence of events may have taken place during the Eemian interglacial or else in the Holocene, so long as a glacial advance took place during the latter after the eruptions had ceased. See text for description of the events depicted

It has been suggested that the Varða tuff cone may be one of a series of small subaerial basaltic pyroclastic cones located on a fissure-erupted chain with a NNE–SSW orientation (Thorarinson 1958). However, the northernmost feature included on the putative fissure is a highly eroded lava outcrop not a cone, and no other volcanic fissures have been reported on Öræfajökull. Additionally, other than the alignment of the Varða tuff cone with two small scoria cones situated c. 0.8 and 1.3 km to the NNE (Fig. 2), there are no associated surface fractures or fault-related scarps that might confirm the presence of a fissure. However, the suggestion from this study of a likely subglacial eruption upslope and potentially along-trend of a line connecting the Varða tuff cone and the two small scoria cones (Figs. 2 and 14) is consistent with the presence of a volcanic fissure. If present, the eruptions did not take place simultaneously along the fissure but occurred on at least three occasions, possibly during the same broad volcanic event but also possibly separated by years (perhaps decades). If they are all related to the same eruptive episode, the slightly different vent locations and different eruptive conditions along the same fissure could have created a small early subaerial phase with aggutinate formed at the vent, followed by a second and probably larger sub-ice event, and then a phreatomagmatic phase (at Varða) where magma in part of the fissure intersected a free-flowing aquifer.

This study thus suggests the following sequence of events (summarised in Fig. 14): (A) Subaerial eruption of two agglutinate cones, probably on a fissure. (B) Subglacial eruption under an inferred Öræfajökull ice cap, possibly on a prolongation of the same fissure system inferred in (A). (C) Jökulhlaup associated with the subglacial eruption. (D) Numerous large ice blocks stranded preferentially on the local watershed by the drained jökulhlaup. (E) Subaerial hydromagmatic eruption of Varða from another part of the same fissure system, with magma interacting with groundwater or seawater in subsurface fractures. (F) Erosion by an ice advance and retreat, resulting in the present-day outcrops.

## Conclusions

Unusual circular structures found in lapilli tuffs of a tuff cone tephra blanket were probably formed by the melting of buried ice blocks, which caused the overlying tephra beds to collapse into and fill the voids thus created. Other means of forming the structures (e.g. melting of the snow cores of buried dirt cones) are possible but less plausible and identification as former ice blocks is favoured. However, whatever their precise nature, the buried objects melted to create voids and were thus glacial in origin. The ice blocks were probably formed as a result of a preceding jökulhlaup that may have been triggered by a subglacial eruption. Called ice-melt subsidence structures, they are the sole evidence for that eruption which is preserved today. The location of the subglacial centre is unknown but presumably was within an ice cap situated higher upslope on Öræfajökull. Thus, the distribution of ice and snow-free ground implied by our study would have been broadly similar to present, precluding eruption within a glacial period. For such structures to be created relies on a tuff-forming eruption burying ice blocks generated by a preceding jökulhlaup since ice blocks do not survive long on the surface (typically only a few years, depending on local circumstances). The structures are analogous to kettle holes found much more commonly on proglacial sandurs and in some lahars sourced in ice-capped volcanoes. The ice-melt subsidence structures at Varða are another proxy for recognising an ice-proximal setting for volcanism and their recognition will enhance our ability to reconstruct palaeoenvironments more reliably, as well as for compiling more compete eruptive histories of regions experiencing glaciovolcanic eruptions.

## Electronic Supplementary Material

ESM 1 View looking northwest across the mouth of Bæjargil showing steeply dipping Varða tephra draped across an older cliff face. (JPEG 1147 kb)

ESM 2 **a**–**c** Views showing numerous large ice blocks strewn across Skeidarársandur following the jökulhlaup associated with the 1996 eruption of Gjálp, Vatnajökull, photographed in 1997. Note the angular shapes of the individual blocks, their varied and often large sizes and steep sides. Although these ice blocks are exposed on the sandur, ice blocks also become completely buried during jökulhlaups (see **d**). **d** Kettle hole developing above an ice block completely buried in a lahar deposited by a jökulhlaup from Gígjökull during the 2010 eruption of Eyjafjallajökull (cf. ice-melt collapse pits of Branney and Gilbert 1995); photographed in July that year. The two steep-sided pits seen in the background are more fully developed kettle holes with vertical sides, also associated with completely buried ice. All images courtesy of Andy Russell. (JPEG 13163 kb)

ESM 3 Series of diagrams illustrating the evolution of subsidence in calderas (modified after Acocella 2007). Four empirical stages are recognised, with different surface expression and development of internal structures. The subsidence may stop at any stage depending on the local circumstances. Note the development of surface sagging and the progressive upward growth of reverse and normal ring fractures. In stage 3, normal faults are depicted migrating upward draped by downsagged beds, a relationship broadly similar to that observed in Varða structures 3 and 4 (cf. Figs. 10 and 12c). The lack of a reverse ring fault cutting the surface at Varða may be due to a combination of a very slow strain rate and the ductility of the cohesive, mechanically weak lapilli tuffs, which reacted by downsagging. However, the presence of local breccias at Varða (e.g. in structure 3; Figs. 11 and 12) indicates that strain rates were variable. (JPEG 399 kb)

## References

[CR1] Acocella V (2007). Understanding caldera structure and development: an overview of analogue models compared to natural calderas. Earth Sci Rev.

[CR2] Acocella V, Cifelli F, Funiciello R (2000). Analogue models of collapse calderas and resurgent domes. J Volcanol Geotherm Res.

[CR3] Branney MJ (1995). Downsag and extension at calderas: new perspectives on collapse geometries from ice-melt, mining, and volcanic subsidence. Bull Volcanol.

[CR4] Branney MJ, Gilbert JS (1995). Ice-melt collapse pits and associated features in the 1991 lahar deposits of Volcán Hudson, southern Chile: criteria to distinguish eruption-induced glacier melt. Bull Volcanol.

[CR5] Branney MJ, Kokelaar P (1994). Volcanotectonic faulting, soft-state deformation, and rheomorphism of tuffs during development of a piecemeal caldera, English Lake District. Geol Soc Am Bull.

[CR6] Branney, M.J. and Kokelaar, P. 2002. Pyroclastic density currents and the sedimentation of ignimbrites. Geol Soc Lond Mem(27):143 pp

[CR7] Brock B, Rivera A, Casassa G, Bown F, Acuña C (2007). The surface energy balance of an active ice-covered volcano: Villarrica volcano, southern Chile. Ann Glaciol.

[CR8] Brown RJ, Bonadonna C, Durant AJ (2012). A review of volcanic ash aggregation. Phys Chem Earth.

[CR9] Brown RJ, Branney MJ, Maher C, Davila Harris P (2010). Origin of accretionary lapilli within ground-hugging density currents: evidence from pyroclastic couplets on Tenerife. Geol Soc Am Bull.

[CR10] Burchardt S, Walter TR (2009). Propagation, linkage, and interaction of caldera rting-faults: comparison between analogue experiments and caldera collapse at Miyakejima, Japan, in 2000. Bull Volcanol.

[CR11] Burke MJ, Woodward J, Russell AJ (2010). Sedimentary architecture of large-scale, jökulhlaup-generated, ice-block obstacle marks: examples from Skeidarársandur, SE Iceland. Sediment Geol.

[CR12] Carey RJ, Houghton BF, Thordarson T (2010). Tephra dispersal and eruption dynamics of wet and dry phases of the 1875 eruption of Askja volcano, Iceland. Bull Volcanol.

[CR13] Cocksedge JE, Eyles N (1983). Road construction in glaciated terrain. Glacial geology.

[CR14] Cole PD, Guest JE, Duncan AM, Pacheco J-M (2001). Capelinhos 1957-1958, Faial, Azores: deposits formed by an emergent surtseyan eruption. Bull Volcanol.

[CR15] Druitt TH (1996). Pyroclastic density currents. Geol Soc Lond, Spec Publ.

[CR16] Fay, H. 2002a. Formation of kettle holes following a glacial outburst flood (jökulhlaup), Skeidarársandur, southern Iceland. In: Snorrason, A., Finnsdóttir, H.P. and Moss, M.E. (eds) The extremes of the extremes: extraordinary floods. Proceedings of the International Association of Hydrological Sciences, 271, 205–210

[CR17] Fay H (2002). Formation of ice-block obstacle marks during the November 1996 glacier-outburst flood (jökulhlaup), Skeiðarársandur, southern Iceland. Spec Publ Int Assoc Sedimentol.

[CR18] Forbes AES, Blake S, Tuffen H, Wilson A (2014). Fractures in a trachyandesite lava at Öræfajökull, Iceland, used to infer subglacial emplacement in the 1727-28 eruption. J Volcanol Geotherm Res.

[CR19] Geyer A, Folch A, Martí J (2006). Relationship between caldera collapse and magma chamber withdrawal: an experimental approach. J Volcanol Geotherm Res.

[CR20] Gilbert JS, Lane SJ (1994). The origin of accretionary lapilli. Bull Volcanol.

[CR21] Hauff J (1969). Ash mounds on Deception Island. Br Antarct Surv Bull.

[CR22] Holohan EP, Schöpfer MPJ, Walsh JJ (2011). Mechanical and geometric controls on the structural evolution of pit crater and caldera subsidence. J Geophys Res.

[CR23] Houghton BF, Wilson CJN (1989). A vesicularity index for pyroclastic deposits. Bull Volcanol.

[CR24] Howard KA (2010) Caldera collapse: perspectives from comparing Galápagos volcanoes, nuclear-test sinks, sandbox models, and volcanoes on Mars. GSA Today 20 . doi:10.1130/GSATG82A.1**no. 10**

[CR25] Hubbard A (2006). The validation and sensitivity of a model of the Icelandic ice sheet. Quat Sci Rev.

[CR26] Johnson JS, Smellie JL (2007). Zeolite compositions as proxies for eruptive paleoenvironment. Geochem Geophys Geosyst.

[CR27] Jónsson J (1982). Notes on the Katla volcanological debris flows. Jökull.

[CR28] Krenek LO (1958). The formation of dirt cones on mount Ruapehu, New Zealand. J Glaciol.

[CR29] Loughlin SC (2002). Facies analysis of proximal subglacial and proglacial volcaniclastic successions at the Eyjafjallajökull central volcano, southern Iceland. Geol Soc Lond, Spec Publ.

[CR30] Magnússon E, Pálsson F, Björnsson H, Gudmundsson S (2012). Removing the ice cap of Öræfajökull central volcano, SE Iceland: mapping and interpretation of bedrock topography, ice volumes, subglacial troughs and implications for hazards assessments. Jökull.

[CR31] Maizels JK (1977). Experiments on the origin of kettle holes. J Glaciol.

[CR32] Maizels JK (1992). Boulder ring structures produced during jokulhlaup flows. Origin and hydraulic significance. Geogr Ann Ser A.

[CR33] Manville V, Hodgson KS, Houghton BF, Keys JR, White JDL (2000). Tephra, snow and water: complex sedimentary responses at an active snow-capped stratovolcano, Ruapehu, New Zealand. Bull Volcanol.

[CR34] Marren PM (2005). Magnitude and frequency in proglacial rivers: a geomorphological and sedimentological perspective. Earth Sci Rev.

[CR35] Martí J, Geyer A, Folch A, Gottsmann J, Gottsmann J, Martí J (2008). A review on collapse caldera modelling. Caldera volcanism – analysis, modelling and response. Developments in volcanology, vol. 10.

[CR36] McDonald BC, Shilts WW (1975). Interpretation of faults in glaciofluvial sediments. In Jopling, a.V. And MacDonald, B.C. (eds) glaciofluvial and glaciolacustrine sedimentation. SEPM Spec Publ.

[CR37] Norðdahl H, Ingólfsson Ó, Pétursson HG, Hallsdóttir M (2008). Late Weichselian and Holocene environmental history of Iceland. Jökull.

[CR38] Okubo CH (2014). Brittle deformation and slope failure at the North Menan butte tuff cone, eastern Snake River plain, Idaho. J Volcanol Geotherm Res.

[CR39] Olszewski A, Weckwerth P (1999). The morphogenesis of kettles in the Höfdabrekkujökull forefield, Mýrdalssandur, Iceland. Jökull.

[CR40] Poppe S, Holohan EP, Pauwels E, Cnudde V, Kervyn M (2015). Sinkholes, pit craters, and small calderas: analog models of depletion-induced collapse analyzed by computed X-ray microtomography. Geol Soc Am Bull.

[CR41] Prestvik, T. 1979. Geology of the Öraefi District, southeastern Iceland. Nordic Volcanological Institute Report, No. 7901, 21 pp

[CR42] Prestvik, T. 1985. Petrology of quaternary volcanic rocks from Öræfi, southeast Iceland. The University of Trondheim and Norwegian Institute of Technology, Department of Geology, Report 21, 81 pp

[CR43] Ren G, Li J (2008) A study of angle of draw in mining subsidence using numerical modelling techniques. Electron J Geotech Eng 13F:14 pp

[CR44] Roberts, M. J. and Gudmundsson, M. T. 2015. Öræfajökull volcano: geology and historical floods. In: Pagneux, E., Gudmundsson, M.T., Karlsdóttir, S. and Roberts, M.J. (eds), Volcanogenic floods in Iceland: an assessment of hazards and risks at Öræfajökull and on the Markarfljót outwash plain. Reykjavík: IMO, IES-UI, NCIP-DCPEM, pp. 17–44

[CR45] Roche O, Druitt TH, Merle O (2000). Experimental study of caldera formation. J Geophys Res.

[CR46] Roche O, van Wyk de Vries B, Druitt TH (2001). Sub-surface structures and collapse mechanisms of summit pit craters. J Volcanol Geotherm Res.

[CR47] Rowley PD, Kuntz MA, Macleod NS (1981). Pyroclastic flow deposits. U.S.G.S. Prof Pap.

[CR48] Russell WJ, Brisbin WC (1990). Primary fractures within a tuff cone, North Menan butte, Idaho, U.S.a. J Volcanol Geotherm Res.

[CR49] Russell AJ, Roberts MJ, Fay H, Marren PM, Cassidy NJ, Tweed FS, Harris T (2006). Icelandic jökulhlaup impacts: implications for ice-sheet hydrology, sediment transfer and geomorphology. Geomorphology.

[CR50] Ryane, C., Russell, K., Edwards, B.R. and Porritt, L.A. 2011. Armoured lapilli in glaciovolcanic deposits: origins and implications. American Geophysical Union, Fall Meeting, abstract #V51C-2531

[CR51] Sharma K, Self S, Blake S, Thordarson T, Larsen G (2008). The AD 1362 Öræfajökull eruption, S.E. Iceland: physical volcanology and volatile release. J Volcanol Geotherm Res.

[CR52] Sohn YK (1996). Hydrovolcanic processes forming basaltic tuff rings and cones on Cheju Island, Korea. Bull Volcanol.

[CR53] Sohn YK, Cronin SJ (2012). Ilchulbong tuff cone, Jeju Island, Korea, revisited: a compound monogenetic volcano involving multiple magma pulses, shifting vents, and discrete eruptive phases. Geol Soc Am Bull.

[CR54] Sohn YK, Park KH (2005). Composite tuff ring/cone complexes in Jeju Island, Korea: possible consequences of substrate collapse and vent migration. J Volcanol Geotherm Res.

[CR55] Stevenson JA, McGarvie DW, Smellie JL, Gilbert JS (2006). Subglacial and ice-contact volcanism at the Öræfajökull stratovolcano, Iceland. Bull Volcanol.

[CR56] Sulpizio R, Zanella E, Macías JL, Saucedo R (1998). Deposit temperature of pyroclastic density currents emplaced during the El Chichón 1982 and Colima 1913 eruptions. Geol Soc Lond, Spec Publ.

[CR57] Swithinbank C (1950). The origin of dirt cones on glaciers. J Glaciol.

[CR58] Thomas RME, Sparks RSJ (1992). Cooling of tephra during fallout from eruption columns. Bull Volcanol.

[CR59] Thorarinson, S. 1958. The Öræfajökull eruption of 1362. Acta Naturalia Islandica, 2, 100 pp

[CR60] Thordarson T, Hoskuldsson A (2002) Classic geology in Europe 3. Terra Publishing & Dunedin Academic Press, Edinburgh, Iceland, 200 pp

[CR61] Tómasson H (1996). The jökulhlaup from Katla in 1918. Ann Glaciol.

[CR62] Tweed FS, Russell AJ (1999). Controls on the formation and sudden drainage of glacier-impounded lakes: implications for jökulhlaup characteristics. Prog Phys Geogr.

[CR63] Visale S, Isaia R (2014). Fractures and faults in volcanic rocks (Campi Flegrei, southern Italy): insight into volcano-tectonic processes. Int J Earth Sci.

[CR64] Wadge, G., Boughton, I., Sparks, S. and Newall, C. 1969. Imperial College volcanological expedition to Iceland 1969. Expedition and geological report, 22 p. [unpubl.]

[CR65] Walker, A.J. (2011). Rhyolite volcanism at Öræfajökull volcano, S.E. Iceland—a window on quaternary climate change. University of Manchester PhD thesis, 325 p. [unpublished]

[CR66] Walter TR, Troll VR (2001). Formation of caldera periphery faults: an experimental study. Bull Volcanol.

[CR67] Waltham T, Bell T, Culshaw M (2005) Sinkholes and subsidence. Karst and cavernous rocks in engineering and construction. Springer-Verlag, Berlin, 382 pp

[CR68] White JDL, Houghton BF (2006). Primary volcaniclastic rocks. Geology.

[CR69] Whittaker BN, Reddish DJ (1989). Subsidence. Occurrence, prediction and control. Developments in geotechnical engineering, vol. 56.

[CR70] Zanella E, Sulpizio R, Gurioli L, Lanza R (1998). Temperatures of the pyroclastic density currents deposits emplaced in the last 22 kyr at Somma—Vesuvius (Italy). Geol Soc Lond, Spec Publ.

[CR71] Zanon V, Pacheco J, Pimentel A (2009). Growth and evolution of an emergent tuff cone: considerations from structural geology, geomorphology and facies analysis of São Roque volcano, São Miguel (Azores). J Volcanol Geotherm Res.

