# Supplementary figures and images for: Complex circular subsidence structures in tephra deposited on large blocks of ice: Varða tuff cone, Öræfajökull, Iceland
Source: Bull Volcanol. 2016 Aug 1;78(8):56. doi: 10.1007/s00445-016-1048-x (PMC7175720; doi:10.1007/s00445-016-1048-x)

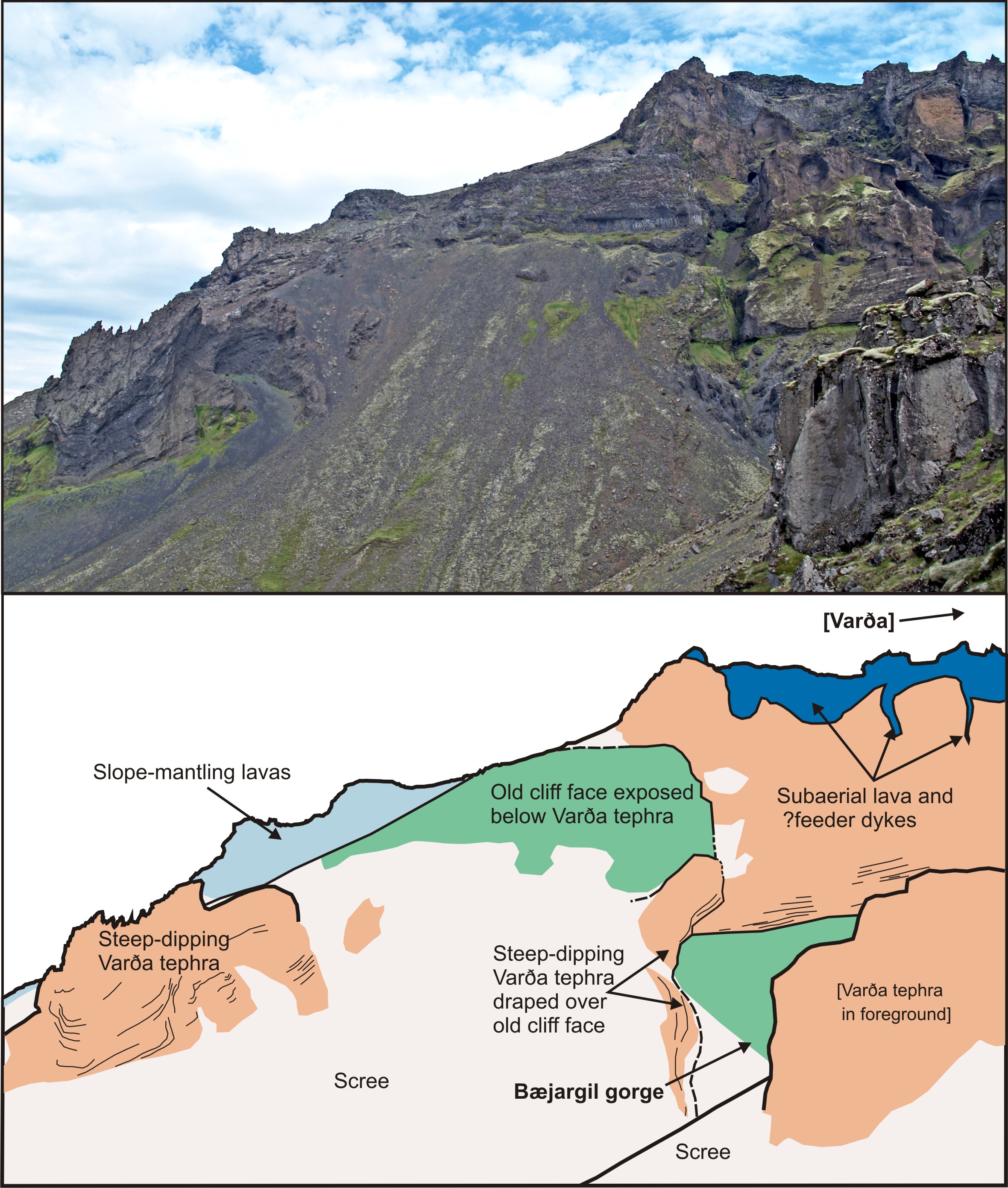

Supplement: Supplementary file 1 — View looking northwest across the mouth of Bæjargil showing steeply dipping Varða tephra draped across an older cliff face. (JPEG 1147 kb) [file 445_2016_1048_MOESM1_ESM.jpg]

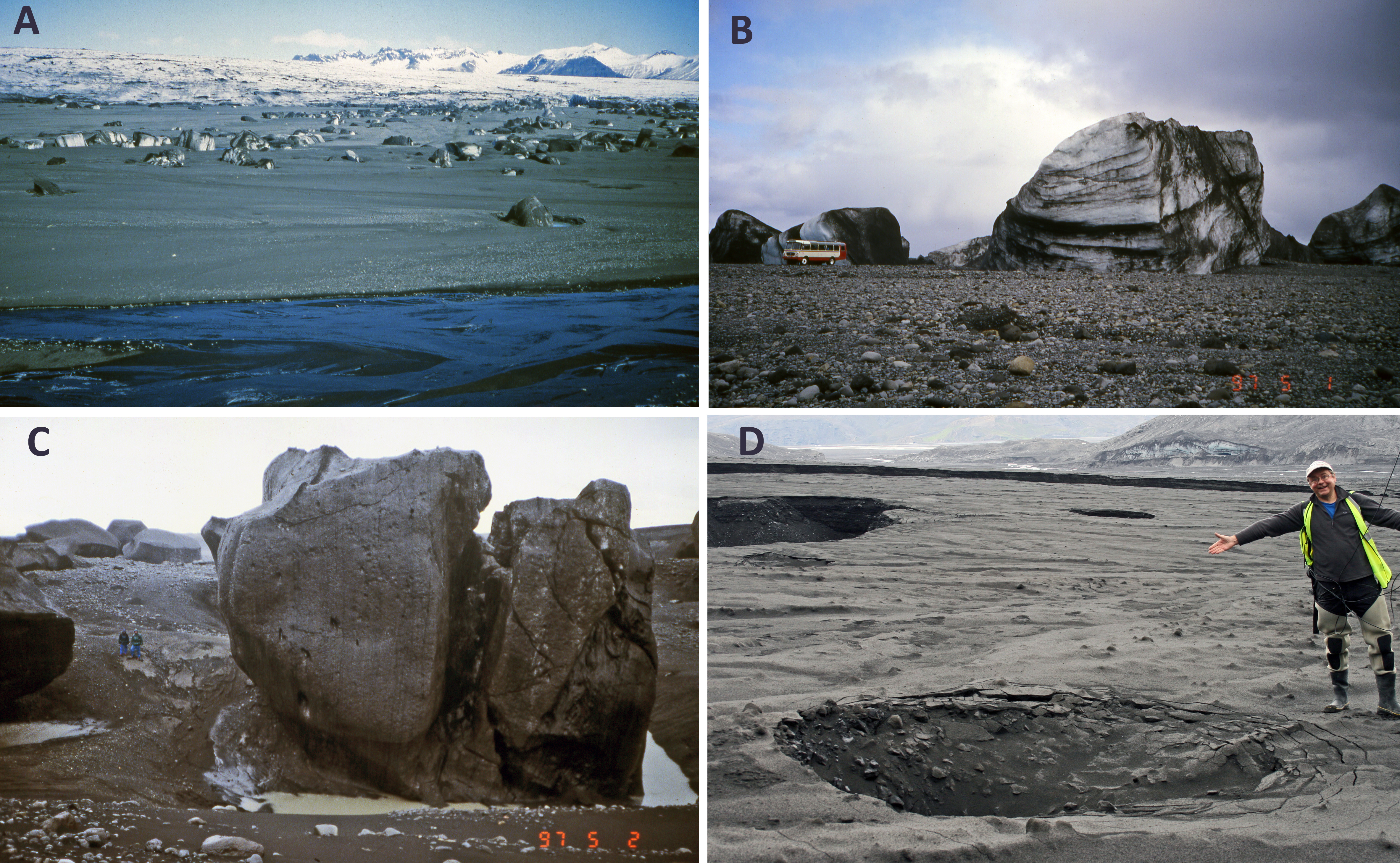

Supplement: Supplementary file 2 — a–c Views showing numerous large ice blocks strewn across Skeidarársandur following the jökulhlaup associated with the 1996 eruption of Gjálp, Vatnajökull, photographed in 1997. Note the angular shapes of the individual blocks, their varied and often large sizes and steep sides. Although these ice blocks are exposed on the sandur, ice blocks also become completely buried during jökulhlaups (see d). d Kettle hole developing above an ice block completely buried in a lahar deposited by a jökulhlaup from Gígjökull during the 2010 eruption of Eyjafjallajökull (cf. ice-melt collapse pits of Branney and Gilbert 1995); photographed in July that year. The two steep-sided pits seen in the background are more fully developed kettle holes with vertical sides, also associated with completely buried ice. All images courtesy of Andy Russell. (JPEG 13163 kb) [file 445_2016_1048_MOESM2_ESM.jpg]

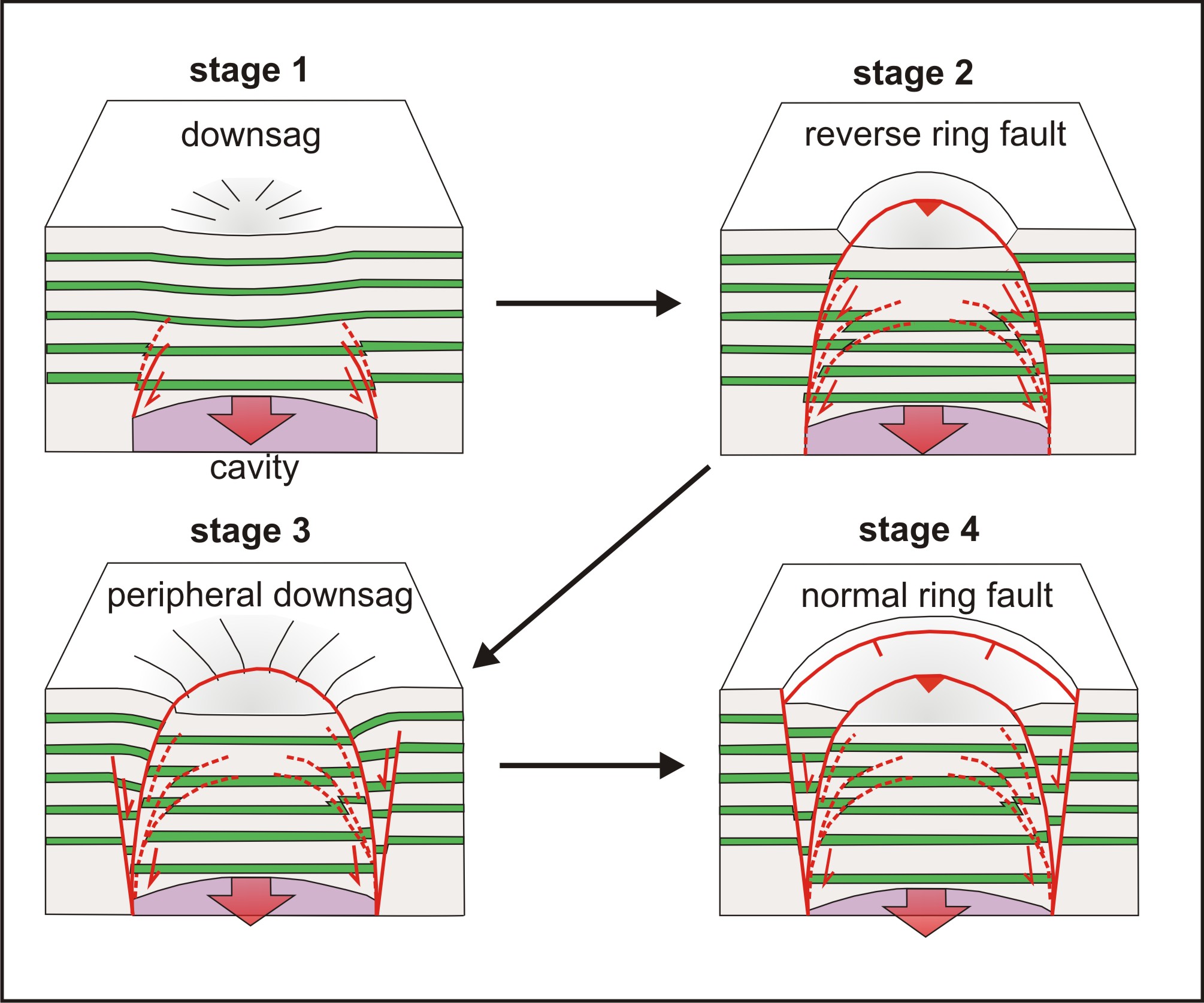

Supplement: Supplementary file 3 — Series of diagrams illustrating the evolution of subsidence in calderas (modified after Acocella 2007). Four empirical stages are recognised, with different surface expression and development of internal structures. The subsidence may stop at any stage depending on the local circumstances. Note the development of surface sagging and the progressive upward growth of reverse and normal ring fractures. In stage 3, normal faults are depicted migrating upward draped by downsagged beds, a relationship broadly similar to that observed in Varða structures 3 and 4 (cf. Figs. 10 and 12c). The lack of a reverse ring fault cutting the surface at Varða may be due to a combination of a very slow strain rate and the ductility of the cohesive, mechanically weak lapilli tuffs, which reacted by downsagging. However, the presence of local breccias at Varða (e.g. in structure 3; Figs. 11 and 12) indicates that strain rates were variable. (JPEG 399 kb) [file 445_2016_1048_MOESM3_ESM.jpg]
